# Contrasted host specificity of gut and endosymbiont bacterial communities in alpine grasshoppers and crickets

**DOI:** 10.1093/ismeco/ycad013

**Published:** 2024-01-10

**Authors:** Florent Mazel, Camille Pitteloud, Antoine Guisan, Loïc Pellissier

**Affiliations:** Department of Ecology and Evolution, University of Lausanne, Lausanne 1015, Switzerland; Département de la mobilité, du territoire et de l‘environnement, Service des forêts, de la nature et du paysage, Sion 1950, Switzerland; Ecosystems and Landscape Evolution, Department of Environmental Systems Science, ETH Zürich, Zürich 8092, Switzerland; Swiss Federal Research Institute WSL, Birmensdorf 8903, Switzerland; Department of Ecology and Evolution, University of Lausanne, Lausanne 1015, Switzerland; Institute of Earth Surface Dynamics, University of Lausanne, Lausanne 1015, Switzerland; Ecosystems and Landscape Evolution, Department of Environmental Systems Science, ETH Zürich, Zürich 8092, Switzerland; Swiss Federal Research Institute WSL, Birmensdorf 8903, Switzerland

**Keywords:** microbiome, microbiota, gut, orthopterans, insect, host specificity, phylosymbiosis

## Abstract

Bacteria colonize the body of macroorganisms to form associations ranging from parasitic to mutualistic. Endosymbiont and gut symbiont communities are distinct microbiomes whose compositions are influenced by host ecology and evolution. Although the composition of horizontally acquired symbiont communities can correlate to host species identity (i.e. harbor host specificity) and host phylogeny (i.e. harbor phylosymbiosis), we hypothesize that the microbiota structure of vertically inherited symbionts (e.g. endosymbionts like *Wolbachia*) is more strongly associated with the host species identity and phylogeny than horizontally acquired symbionts (e.g. most gut symbionts). Here, using 16S metabarcoding on 336 guts from 24 orthopteran species (grasshoppers and crickets) in the Alps, we observed that microbiota correlated to host species identity, i.e. hosts from the same species had more similar microbiota than hosts from different species. This effect was ~5 times stronger for endosymbionts than for putative gut symbionts. Although elevation correlated with microbiome composition, we did not detect phylosymbiosis for endosymbionts and putative gut symbionts: closely related host species did not harbor more similar microbiota than distantly related species. Our findings indicate that gut microbiota of studied orthopteran species is more correlated to host identity and habitat than to the host phylogeny. The higher host specificity in endosymbionts corroborates the idea that—everything else being equal—vertically transmitted microbes harbor stronger host specificity signal, but the absence of phylosymbiosis suggests that host specificity changes quickly on evolutionary time scales.

## Introduction

Macroorganisms are sometimes colonized by dense microbial populations that can provide key functions to their hosts [1, 2]. Classic examples of these associations include mutually beneficial symbiosis between aphids and proteobacteria *Buchnera aphidicola* [3] or the Hawaiian bobtail squid and bioluminescent *Vibrio fisheri* [4]. These biological alliances are often relatively simple and highly specific: a given host species associates only with a specific microbial partner and vice versa [2]. However, the extent to which these examples of strict and relatively simple beneficial symbiosis are representative of the natural diversity of associations between micro and macroorganisms is debated [5-9]. The recent developments of DNA metabarcoding and metagenomic approaches have revealed complex situations where macroorganisms inner and outer surfaces are colonized by diverse communities of microbes, forming systems where one host associates with multiple symbionts (systems with 1 host and n symbionts) [10-13]. Although the composition of these complex communities is influenced by host ecology, it is also often related to host identity and host phylogeny, i.e. harbor “phylosymbiosis” [14]. Phylosymbiosis is a special case of the broader concept of “host specificity” developed in the parasitology [15] and mutualism [2] literature. Traditionally, host specificity has been quantified at the scale of individual symbiont members, for example as the number of host species (host range) that are colonized by the symbiont, but can incorporate or not host phylogenetic relationships [16]. The host specificity concept at the individual symbiont scale can be conceptually extended to an entire community of symbionts, as the degree to which a particular host lineage associate with a compositionally distinct symbiont community [17]. Here, we distinguish between two types of host specificity. First, “phylosymbiosis” is defined as a significant correlation between microbiota composition and host phylogeny (i.e. closely related host species harbor more similar microbiota than distantly related host species) [14]. Second, microbiota-level host *species* specificity (“host specificity” for simplicity hereafter) is defined as a significant correlation between microbiota composition and host species identity (i.e. individual hosts from the same species harbor microbiota with more similar composition than individual hosts from different species).

A range of non-mutually exclusive mechanisms can foster both host specificity and phylosymbiosis [18] or the lack thereof when those conditions are not met. First, the mode of microbial transmission across hosts can determine the conservatism of microbiome clade across hosts [17, 19]. Theory predicts that—everything else being equal—vertical transmission should foster host specificity and phylosymbiosis [18, 20], and data confirm this prediction: a more vertical transmission mode correlates with higher specificity in mammals [17]. Second, the host can “control” microbial composition via antimicrobial compounds or rewards [21, 22]. Third, phylogenetically conserved ecological traits of the hosts can indirectly select (filter) the composition, for example, diet [23]. The two last mechanisms share similarities with the filtering concept developed in community ecology: they both consider host species as a singular habitat only colonizable by a restricted subset of microbes from a larger pool [24-26]. However, they fundamentally differ from an evolutionary perspective. The host “control” mechanism is assumed to have evolved as a way to regulate microbial colonization and prevent the invasion of cheaters [21, 22], for example through the production of antimicrobial peptides by the immune system [27, 28]. In contrast, the “by-product” filtering mechanism is mediated by an host trait that did not necessarily evolved to control microbial populations [8, 9, 23], for example diet [29], gut oxygen level, or gut pH [30]. Although experimental tests of these theories have provided valuable insights into the underlying mechanism of host specificity and phylosymbiosis [31, 32], large-scale *in situ* analysis of wild macroorganisms is needed to provide general conclusions. In particular such studies have revealed that the degree of host specificity and phylosymbiosis varies widely between types of microbiota (e.g. external versus internal) and the identity of the host and microbes [20, 23]. For example, non-volant mammals harbor strong phylosymbiosis signal [14, 33-35] in contrast to birds or bats [11]. This natural variation of host specificity and phylosymbiosis across systems represents an important but overlooked source of data that offers a unique opportunity to explore the mechanisms behind host specificity and phylosymbiosis [20, 23]. Since most studies have focused on a limited and biased set of host lineages and host habitats—mainly mammalian guts, we currently lack a good understanding of the prevalence and strength of host specificity and phylosymbiosis across most macroorganisms. Recent studies on other taxonomic groups have challenged the idea that phylosymbiosis in animal associated microbiomes is a pervasive pattern [5, 13]. For instance, a recent massive study measured the strength of host-microbiota phylosymbiosis across 1000 microscopic marine invertebrates from 21 phyla and found no signal of phylosymbiosis [13]. The relationships between microbial composition and host evolutionary history should be explored across more taxonomic groups.

Arthropods represent an excellent system to measure the strength of host-microbiome specificity and phylosymbiosis in nature because they are widespread, species-rich, and harbor multiple distinct microbiota within or in contact to host tissues (e.g. endosymbionts within tissues and gut or cuticle symbionts) that offer an opportunity to contrast their host specificity and phylosymbiosis signals [36]. Arthropods are known to associate with endosymbionts, and their microbiota includes species in the genera *Wolbachia*, *Cardinium*, *Rickettsia*, and *Spiroplasma*, which colonize the host’s body [37]. This microbiota is usually relatively simple (low species richness, even sometime one single strain, i.e. a 1 host-1 symbiont system), can engage in intimate relationships with its host, sometimes manipulating its reproduction, intraspecific, and interspecific communication, and harbor high degree of host specificity [38, 39]. In contrast, the arthropod gut microbiota is a more diverse community that can be acquired and influenced by the environment [40] and may perform multiple function for its host in some case [41] but not others [42]. The arthropod gut microbiota host specificity and phylosymbiosis is highly variable across host clades, being for example high in bees [43] but low in spiders [36] and generally understanding of its composition remains limited.

Here, we document the strength of host specificity and phylosymbiosis in alpine orthopterans (grasshoppers and crickets), a group of generalist herbivorous and omnivorous insects in the Swiss Alps. We sampled guts of 336 individuals from 24 species across a large elevational and environmental gradient (601-2277 masl, Supplementary Fig. 1) and used metabarcoding and amplicon sequencing (partial 16S rDNA) to measure and contrast microbiome composition within and across host lineages. We hypothesize that: (i) both endosymbionts and putative gut symbionts communities harbor host specificity and phylosymbiosis but that (ii) endosymbionts should harbor stronger host specificity and phylosymbiosis signal due to their more intimate relationship with the host and vertical transmission between generations and that (iii) gut symbionts should be more influenced by the environment (here elevation) and geography.

**Figure 1:**
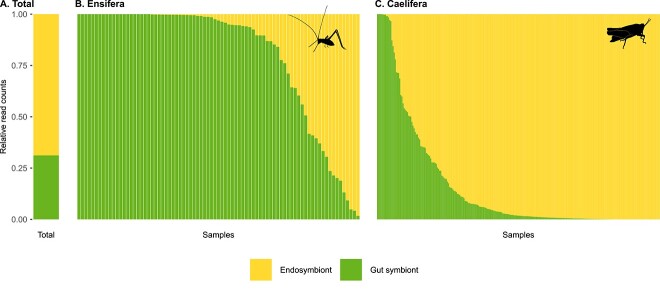
Relative read counts of endosymbionts and putative gut symbionts across samples; stacked bar plot depicts the relative read counts (Y-axis) of either endosymbionts (*Spiroplasma* + *Wolbachia*) or putative gut symbionts ASVs across samples (X-axis, *n* = 336); total relative read counts across all samples (panel A) or values per sample in different host lineages (1 stacked bar = 1 sample, panel B–C) are given; silhouettes from Birgit Lang available from phylopic.org.

## Material and methods

### Sampling and wet lab

####  

##### Animal sampling

Animals were sampled in nine sites of the western Swiss Alps (Supplementary Fig. 1, Supplementary Tables 1 and 2) during summer 2017 using hand net. We collected 368 specimens for 24 species, sampling 2–3 specimens per species present, per sex, and at each site (metadata provided in Supplementary Table 2 and summary of the sampling size per sites, species, and sex provided in Supplementary Table 3). Animals were euthanized by freezing and conserved at −20°C until dissection. The sampling complies with national regulation (Swiss Permit number #2364). Details concerning location of sampling sites can be found in Supplementary Table 1.

### DNA extraction

Orthoptera gut samples were entirely extracted from preserved specimens through dissection under sterile conditions and conserved at −20°C. DNA was extracted from gut samples using DNeasy® PowerSoil® HTP 96 Kit (Qiagen, Hilden, Germany) following manufacturer protocol. DNA was conserved at −20°C following extraction.

### Amplicon sequencing

A genetic marker was amplified using the 16 s primer pair 341f/785r (341f-16 s: CCTACGGGNGGCWGCAG—18 nt; 785r-16 s: GACTACHVGGGTATCTAATCC—21 nt) that generate a fragment of 444 bp [44]. The Polymerase Chain Reaction (PCR) mix was composed of 4 μl of DNA extract, 11 μl of KAPA HiFi HotStart ReadyMix (Roche), and 5 μl of each primer at 1 μM (MicroSynth, Balgach, Switzerland). The PCR reactions started with a denaturation step at 95°C for 3 min followed by 32 cycles of 95°C for 30 s, 55°C for 30 s, and 72°C for 30 s, and terminated with an elongation step of 72°C for 5 min. After a purification step with ethanol DNA precipitation, 1 μl of cleaned PCR products were used in a ligation PCR using Nextera Illumina i5/i7 indices. Libraries were pooled in equimolar ratio and sequenced on a MiSeq Illumina platform (AIMethods, Leipzig, Germany). Corresponding sequences are publicly available on ENA (PRJEB62030), and metadata are provided in Supplementary Table 2.

### Sequence processing

Primers from raw sequences were first trimmed using Cutadapt 4.4 [45] with the following parameters: e = 0.1, m = 100. Trimmed sequences were then processed using the dada2 R package [46]. Reads were quality-filtered using the *filterAndTrim* dada2 R function (with parameters maxEE = 4, truncQ = 2, truncLen = 260), merged using the *mergePairs* function, and chimeras were removed using the *removeBimeraDenovo* function. We assigned taxonomy for each amplicon sequence variant (ASV) using the naïve Bayesian RDP classifier [47], as implemented in dada2 (function *assignTaxonomy*, parameter minBoot set to 60) with the SILVA (version 138) database [48]. We removed all ASVs not assigned to an Order or assigned to mitochondria or chloroplast and with length < 390 nucleotides. The final count of ASV was 1957. We only kept samples with more than 1000 reads for subsequent analysis (*n* = 336 samples).

### Host phylogeny reconstruction

The host phylogeny was produced using COI, COII, CytB, and 16s genes retrieved from Genbank and completed with COI custom sequencing data and unpublished data from colleagues. Custom sequences are deposited on figshare (10.6084/m9.figshare.23605404), and GenBank accession numbers are provided in Supplementary Table 4. Sequences were aligned through multiple alignment using a Geneious algorithm [49] with a cost matrix of 93% similarity threshold. Alignments of each marker were concatenated, and the phylogeny was generated using the RaxML program [50] on the CIPRESS portal [51]. Details on the method for the phylogeny reconstruction and the produced tree are given as supplementary text (Supp. Information).

### Amplicon sequence variant phylogeny reconstruction

ASVs sequences were aligned with mafft (v7.490) [52] using default parameters. Phylogeny was then inferred using FastTree (V. V2.1.11) [53] with the GTR + CAT model. As *Wolbachia* was the most abundant lineage found in the dataset and to better identify which *Wolbachia* lineages are present in alpine orthopterans, we reconstructed a phylogeny restricted to the ASVs assigned to this genus. As short amplicon sequences are known to contain only few informative sites for phylogenetic reconstruction, we guided the ASV phylogenetic reconstruction using a backbone phylogeny. We derived this backbone phylogeny from full 16S sequences of a representative set of *Wolbachia* lineages representing major defined “super groups” [54]. Eighty-six genome assemblies of the representative lineages were retrieved from the NCBI website following the method described by Kaur *et al.* [54], and 16S sequences were extracted with barnap v0.9 with default parameters (https://github.com/tseemann/barrnap), filtered to keep only sequences >1200 pb and aligned using mafft. A maximum likelihood phylogeny was reconstructed using IQTREE v1.6.12 [55]. The TIM3 + F + G4 model evolutionary model was selected based on Bayesian info criterion (option -m TEST) [56]. We used the backbone full 16S alignment to constrain the alignment of the partial 16S ASVs sequences using mafft (with options—addfragments—keeplength). Finally, an ASV phylogeny was constructed using IQTREE where the backbone phylogeny was used as constrains (using the—g option). The TN + F + G4 evolutionary model was selected based on Bayesian info criterion. We quantified the robustness of each node using ultrafast bootstrap (*n* = 1000) [57].

### Statistical analysis

ASVs were defined as endosymbionts or putative gut symbionts based on their taxonomic assignation: all ASVs assigned to the genus *Wolbachia* and *Spiroplasma*, or assigned to the order *Rickettsiales* or *Chlamydiales* were defined as endosymbionts, and all the remaining ASVs were classified as putative gut symbionts.

We used a Kruskal–Wallis test (function *kruskal.test* in R) to test whether Caelifera (grasshoppers) and Ensifera (crickets) hosted a different relative read counts of endosymbionts. We used ANOVA to test (within each of these host lineages) whether different sex and different species host different relative read counts of endosymbionts (univariate models). All subsequent analysis was run in parallel for endosymbionts and putative gut symbionts communities. Alpha-diversity was estimated using the Chao1 index. Beta-diversity was primarily estimated using Bray–Curtis metric using a rarefied table (*n* = 1000 reads per sample). We represented dissimilarity between samples using Non-metric Multidimensional Scaling (NMDS) with two axes and, for ease of representation, we excluded samples from the NMDS and the corresponding tests if they hosted a unique ASV that was only found in this sample (*n* = 2 for putative gut symbionts and *n* = 4 for endosymbionts) as these sampled cannot be adequately placed in the compositional space. We tested for the effect of host species, sex, and elevation on beta-diversity using Permutational Analysis of Variance (PERMANOVA) (function *adonis2* in vegan, *n* = 999 permutations) with marginal sums of squares [58] and using omega^2^ as a measure of effect size (omega^2^ is equivalent to adjusted R^2^, i.e. R^2^ adjusted for the number of predictors). We note that other methodological approaches to quantify specificity, such as Bayesian mixed models are being developed and deserve careful consideration in future studies [57] . PERMANOVA can confound location and dispersion effects if there is significant dispersion in the data [59]. Using the vegan R function *betadisper*, we measured and found significant dispersion for the effect of host species (F tests using the R function *anova*, *P* < .05). To test whether the PERMANOVA results are not only driven by dispersion effects, but also location effects, we re-run PERMANOVA with a balanced design for host species (i.e. equal number of samples for each host species) as recommended by Anderson and Walsh [59]. To do so, we selected 15 host species with at least 4 individual each and with >1000 endosymbiont reads, we randomly selected 4 individuals in each species and performed a PERMANOVA. We repeated the procedure 100 times and report median statistics (pseudo-F, R^2^ and *P*-value). We also tested the robustness of our results to (a) the rarefaction step by running our beta-diversity analysis without rarefaction and (b) the beta-diversity metric using the Jaccard (with presence/absence), the UniFrac, and the Aitchison metric as an alternative. We also tested whether the composition of individual endosymbiont lineages correlated with host species identity by running a PERMANOVA test for *Wolbachia* and *Spiroplasma* independently (Bray–Curtis dissimilarity metric, data rarefied to 500 reads per sample). To quantify phylosymbiosis, we followed Mazel *et al*. [23] and measured correlation between phylogenetic distance and microbial compositional dissimilarity using a Mantel test (n permutations = 999, Bray–Curtis and Jaccard dissimilarity, data rarefied to 1000 reads/sample). To avoid pseudo-replications due to multiple individuals per species, we averaged inter host species dissimilarities [23]. We also measured phylosymbiosis incorporating phylogenetic relationships between the ASVs by using weighted UniFrac dissimilarity metric [60].

To further test the effect of elevation on microbiome composition within host species, we selected two hosts’ species that were sampled along a wide elevational range (*Chorthippus parallelus* and *Euthystira brachyptera*). As each elevation was represented by only one site and to avoid confounding site and elevation effects, we used the following strategy. We took the median beta-diversity values between sites and carried ordinations and PERMANOVA test of elevation effects on these inter-site dissimilarity values.

To evaluate the correlation contribution of individual ASV to the phylosymbiosis signal, we built random forest models using the “randomForest” function in the R package randomForest (https://www.stat.berkeley.edu/~breiman/RandomForests/). We built 100 classification trees (the response variable being the host species identity and the explanatory variables being the ASV distributions across samples) and assessed the significance of the out of bag error rate by performing 99 randomizations of the data by shuffling host identity across samples. We evaluated the contribution of each ASV to the global model using the function “importance” in the R package randomForest. Briefly, each predictor variable (here the distribution of each individual ASV) is randomized, and the fit of the global model is then compared to the non-randomized model. The importance score of each ASV is measured as the decrease in the Gini index of node impurity between the non-randomized and randomized model.

### Data availability and reproducibility of the study

All the bioinformatic pipeline described above has been written in BASH and R with use of the tidyverse [61], vegan [62], phyloseq [63], and ggplot [64] R packages. The associated R code is publicly published on *github* (https://github.com/FloMazel/orthopteran-microbiome). Metadata have been formatted following the Minimum information about any Sequence (MIxs) standard and is provided as Supplementary Table 2. Sequence data are available at ENA website under the project ID PRJEB62030 (microbes 16S sequences), on figshare (DOI: 10.6084/m9.figshare.23605404, host DNA sequences), and host Genbank accession numbers are provided in Supplementary Table 4.

## Results

Overall, endosymbionts belonging to the genera *Wolbachia* and *Spiroplasma* represented the majority of 16S reads across the 336 samples (65%, Fig. 1A). They were also widespread across host species: *Wolbachia* was found at >10% relative read counts in at least one individual in 79% of the host species (19/24), while *Spiroplasma* was found in 75% of the host species (18/24). Phylogenetic reconstruction of *Wolbachia* ASVs suggests that the recovered sequences belong to *Wolbachia* supergroups A, B, and F (Supplementary Fig. 2). In contrast, putative gut symbionts represented a lower portion of 16S reads (35%, Fig. 1A) and belonged to families *Enterobacteriaceae*, *Erwiniaceae* (notably from the genus *Pantoea*), *Sphingomonadaceae*, and *Streptococcaceae* (Supplementary Fig. 3).

The relative proportion of read counts of endosymbionts vs. putative gut symbionts largely differed between crickets (suborder Ensifera, generally omnivores) and grasshoppers (suborder Caelifera, generally herbivores). For grasshoppers, 84% (sd +/−27%) of the reads originated from endosymbionts, while for crickets, this dropped to 20% (sd +/−30%, Fig. 1B and C, Kruskal–Wallis chi^2^ = 132.4, *n* = 336, *P*-value <0.01). For both crickets and grasshoppers, the proportion of endosymbionts reads varied significantly between host species (Supplementary Fig. 4 and Supplementary Table 5, *P*-value Kruskal-Wallis (KW) test <.05), the interaction between sex and species was found significant in grasshoppers (Supplementary Fig. 4 and Supplementary Table 5).

**Figure 2:**
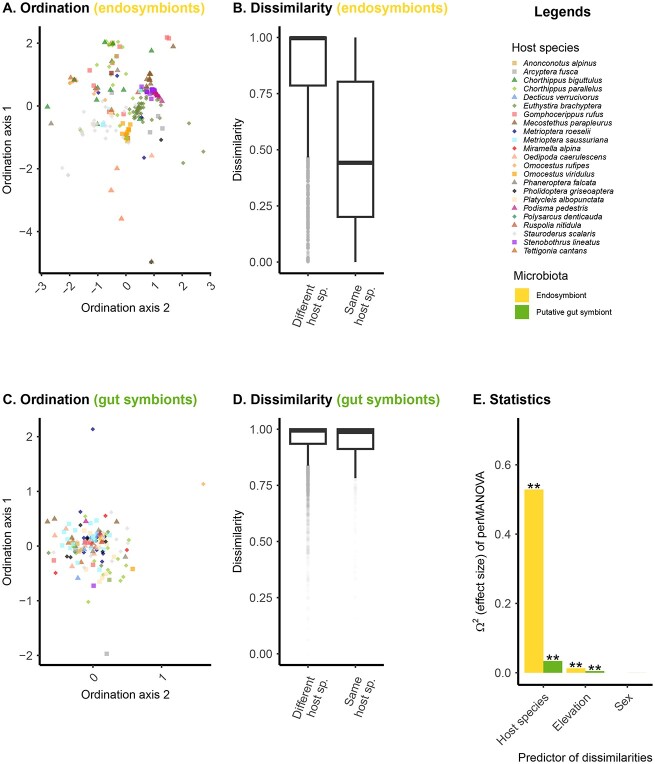
Host specificity of endosymbiont and putative gut symbiont communities; the figure illustrates (panels A–D) and report statistical measures (panel E) of host specificity; panel A and C are multidimensional representations of microbiome composition (NMDS axes based on Bray–Curtis dissimilarities between samples, see alternative metrics in Supplementary Figs 11–13) for endosymbiont (panel A) and putative gut symbiont communities (panel C); panel B and D display values of microbiome compositional dissimilarities between pairs of samples from the same or different host species (endosymbiont in panel B and putative gut symbiont communities in panel D); panel E depicts the strength of the effect (Y-axis) of different host factors (X-axis) on microbiota composition (PERMANOVA model on beta-diversity); the “host species” effect measures the strength of host specificity at the species level, and the asterisk refers to the level of significance of the corresponding factor in the PERMANOVA model.

**Figure 3:**
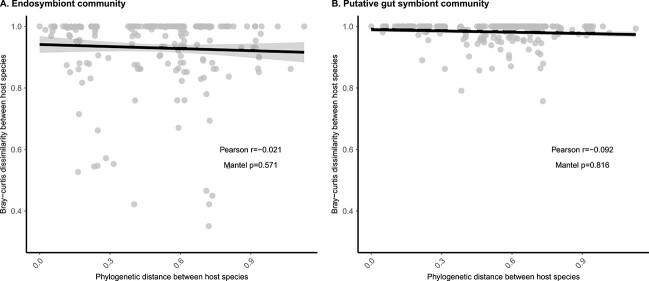
Non-detectable phylosymbiosis of endosymbiont and putative gut symbiont communities; the figure depicts the relationship between microbiota dissimilarity (Bray–Curtis measure) and host phylogenetic distance (X-axis) for endosymbionts (panel A) and putative gut symbiont (panel B) communities; a given point represents a unique pair of host species, and Bray–Curtis dissimilarity value between host pair is calculated as the average Bray–Curtis dissimilarity across all pairs of individuals belonging to the two species; Mantel *P*-value are based on 999 permutations.

**Figure 4:**
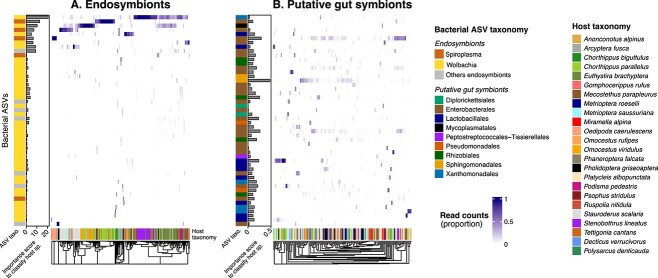
Bacterial ASV distribution across samples and host species; the figure depicts the distribution of individual ASVs (rows) across samples and hosts (columns) for endosymbionts (panel A) and putative gut symbiont (panel B) communities; bacterial ASVs taxonomy is shown on the left side of the heatmap, while host taxonomy is given on the bottom of the heatmap; important score for each ASV to classify sample to host species (random forest models) is given as a barplot on the left side of each heat map; only the top 50 most abundant endosymbiont and putative gut symbionts are represented.

As endosymbionts and putative gut symbionts are likely located in separated host compartments and develop a very different relationship with their host, we analyzed their community structure (i.e. richness and composition) independently. For each microbiome types (endosymbionts and putative gut symbionts), we only retained samples with at least 1000 DNA reads. We kept 232 samples for the endosymbionts analysis and 145 samples for putative gut symbiont analysis, with 76 samples shared between the two data subsets. On average, endosymbionts exhibited lower richness than putative gut symbionts: 4.5 ASVs/samples vs. 12.5 ASVs/Sample (Supplementary Fig. 5) with one main dominant endosymbiont ASVs in each sample: the most abundant *Spiroplasma* (resp. *Wolbachia*) ASV grouped on average 99% (resp 79%) of the reads in each sample (Supplementary Fig. 6). Prevalence of ASVs across samples was relatively low (4.8 and 2.3 samples on average for endosymbionts and putative gut symbiont, respectively, Supplementary Fig. 7). Host specificity and phylosymbiosis were quantified by measuring the strength of the correlation between (1) microbiota composition and host species identity and (2) microbiota composition and host species phylogeny. We found that both types of microbiota showed signal of specificity at the host species level: the microbiota of individuals from the same host species were more similar than individuals from different host species (i.e. composition clustered by host species, PERMANOVA: *P*-value <.05, Fig. 2). We did not detect specificity at the scale of the host phylogeny (phylosymbiosis), i.e. closely related host species did not host microbiota more similar than distantly related hosts (Mantel test, *P*-value >.05, Fig. 3, Supplementary Fig. 8 for host phylogeny). At the host species level, we found that the strength of specificity, as quantified by omega^2^ (equivalent to adjusted R^2^) was higher (~ 5 times, Fig. 2E) in endosymbionts (omega^2^ = .53, pseudo-F = 15.6, *n* = 260, Fig. 2A and B) than for putative gut symbionts (omega^2^ = .04, pseudo-F = 1.22, *n* = 41, Fig. 2C and D). This host specificity signal was also observed for *Wolbachia* and *Spiroplasma* independently (Supplementary Fig. 9). With regard to environmental and biological factors, we found that elevation, but not sex, correlated to microbiota composition (Fig. 2E). These results were robust to various methodological choices, including rarefaction (Supplementary Fig. 10), beta-diversity metric, notably the Jaccard metric (presence/absence) data, Supplementary Fig. 11), the Aitchison metric that is robust to the compositionality aspect of the data (Supplementary Fig. 12), and the UniFrac metric, which takes into account phylogenetic relationships between symbionts (Supplementary Fig. 13). Additionally, permutation procedures were employed to account for sampling site effects (Supplementary Fig. 14) and dispersion effects (Supplementary Table 5). Mantel results were also robust to the beta-diversity metric used (Supplementary Figs 15–17). We further confirmed the effect of elevation on microbiota composition, by selecting two species that were sampled along a large elevational gradient (*Chorthippus parallelus* and *Euthystira brachyptera*). We found that change of composition within host species across sites was related to elevation of the sites for endosymbionts, but not for putative gut symbionts (Supplementary Fig. 18 for Bray–Curtis beta-diversity metric, Supplementary Fig. 19 for Aitchison beta-diversity metric). Overall, we note that our findings are robust to compositionally aware beta-diversity metrics as well as more classical metrics. Next, we explored which ASVs contributed most to the host specificity signal observed at the community level using random forest: models performed better for endosymbionts than for gut symbionts, in agreement with the beta-diversity analysis (out of bag error = 28% and *P* < .01; out of bag error = 86% and *P* = .1, for endosymbionts and gut symbionts respectively, Supplementary Fig. 20). We found that some endosymbionts—both Wolbachia and *Spiroplasma*—contributed disproportionally to the host-specificity pattern as they were restricted to only one or a few host species (Fig. 4, panel A, importance score per ASV show in the left bar plots). This stands in stark contrast with putative gut symbionts that were poor classifier of host species (Fig. 4, panel B, importance score per ASV show in the left bar plots).

## Discussion

The microbiome of insects is influenced by a combination of ecological and evolutionary factors. Here, we performed a large-scale characterization of gut-associated microbial communities of alpine orthopterans (grasshoppers and crickets) in the Swiss Alps by sampling the guts of 336 individuals from 24 species across a large elevation gradient. We showed that the microbiome composition cluster by host species but does not correlate to host phylogeny. Our results highlight the importance of the host ecology, including elevation and geography, in determining microbiota composition, but an absence of phylosymbiosis.

For both endosymbionts and putative gut symbionts, we found that individuals from the same host species harbored microbiota with more similar composition than individuals from different species. Similarity among conspecific individuals can arise because of several non-mutually exclusive mechanisms [18] including the mode of microbial transmission across hosts [17, 19], a “control” by the host [21, 22], and “by-product” filtering by the host [23]. Theory suggests that this “by-product” filtering mechanism represents a plausible model and a good default (or “neutral”) expectation when patterns of specificity are weak because it does not rely on complex microbiota-host dialogue and selection of an active “control” mechanism by the host [23]. Here, given weak specificity signal recovered for putative gut symbionts and given theoretical result showing that week symbiosis can be produced by a “by-product” mechanism alone [23], we suggest that “by-product” filtering is the most plausible mechanism to explain the pattern of host specificity. However, we acknowledge that further experimental studies will be essential to test which of these two alternative theories most likely apply to the gut microbiome. Multiple host traits could mediate this mechanism and include diet, habitat and elevation, but the elevational effect is difficult to disentangle from geographical effects as we sampled animals across one elevational gradient so that sites that are more similar in elevation are also closer in space. Further studies could sample microbiome along several independent elevational gradient to tease apart elevation from geography. To identify which traits mediate host filtering, further studies could also simultaneously measure putative filtering traits, e.g. host diet, along with the microbiome, and determine whether differences in composition between host species (i.e. host specificity) are driven by differences in host diet [65]. Overall, turnover of putative gut symbionts between individual was very high (Bray–Curtis values > .8) and the explanatory of our beta-diversity models relatively low (omega^2^ < 10%). Although these results are not uncommon in gut microbiome studies, they are compatible with the idea that some of the DNA sequences recovered here could originate from transient microbes (e.g. living on plants) and not from resident gut symbionts that have a positive population growth rate in the gut environment [5].

Specificity to host species was ~5 times stronger for endosymbionts than for putative gut symbionts. This is particularly obvious when comparing ASV sharing within and between host species (Fig. 2B and D). Interestingly, we found that this finding was driven by a few endosymbiotic ASVs (both Wolbachia and *Spiroplasma*) that are highly specialists to their host and can be used to classify host species identity in random forest models. This difference in host specificity corroborates the idea that microbes engaging in a more intimate relationship with their host are also more specific to them. We suggest this could be mediated by the vertical mode of transmission of endosymbionts that contrasts with the mixed mode of putative gut symbionts transmission, i.e. horizontal and vertical [66-68]. Indeed, *Wolbachia* and *Spiroplasma* have been shown to be vertically inherited between mother and offspring through colonization of the oocytes, which favors microbial dispersion between conspecific individuals rather than heterospecific individuals and is expected to foster specificity [17, 19]. It will be interesting for future studies to explore in more details the few highly specific ASV we found, for example by reconstructing their genomes using shotgun metagenomic sequencing.

For both endosymbiont and putative gut symbiont communities, we did not recover host specificity at the scale of host phylogeny, a pattern sometimes called “phylosymbiosis” where closely related species harbor more similar microbiota than distantly related species. This implies that, even for endosymbionts, there is a decoupling between host evolutionary history and the composition of its symbiotic communities, even if we cannot totally rule out that the absence of phylosymbiosis in crickets could be due to the contamination of *Wolbachia* strains infecting the host prey. Also, we used an uncalibrated host phylogeny, as it is commonly done, but it would be interesting for future studies to explore the effect of host phylogeny calibration on the detection of phylosymbiosis. This result is in broad agreement with phylogenomic analysis documenting a lack of congruency between host and *Wolbachia* phylogenies or genetic divergences [69, 70] but also with observations that *Spiroplasma* can switch between hosts in the laboratory [71]. Altogether, this suggests that endosymbionts can be easily swapped between host species across evolutionary time (a pattern sometimes referred to as « horizontal transfer »). In *Wolbachia*, these cross-species transfer occurs via multiple mechanisms including feeding on infected plant material [72] or predation and cannibalism. Although the coarse phylogenetic resolution of our amplicon data (440 pb of the V4 region of the 16S rRNA gene) limits our ability to directly test for phylogenetic congruency between host and endosymbionts, the observed lack of phylosymbiosis suggests that endosymbionts distribution across hosts is not dictated by host phylogenetic relationship. This is compatible with a model where endosymbionts can easily switch between closely and distantly related host species. For putative gut symbionts, this absence of phylosymbiosis stands in stark contrast to results in non-volant mammals for example, where phylosymbiosis is prevalent [11]. In mammals, phylosymbiosis is often detected when widely divergent hosts are included, but sometimes disappears or becomes weaker when only closely related hosts are included [73]. This effect of phylogenetic scale on the detectability of phylosymbiosis could arise if phylosymbiosis is shaped by by-product filtering but the host traits that filter microbes did not diverge enough between closely related species (e.g. diet is often largely overlapping between closely related mammals). Here, the phylosymbiosis signal is absent despite selecting broad phylogenetic coverage with divergence between genera within Caelifera or Ensifera ranging from 5 to 95 MyA, roughly similar to studies detecting phylosymbiosis in mammals.

We observed marked differences in endosymbionts relative read counts between two main lineages of hosts: grasshoppers (Caelifera, herbivores) harbored ~5 times more endosymbionts relative read counts than crickets (Ensifera, omnivores). Moreover, we found that grasshoppers disproportionally host endosymbionts and only traces of putative gut symbionts suggesting that—if beneficial function is only provided by an abundant gut microbiome [5]—the putative gut symbionts might not play an important role for their hosts, at least for the populations and species studied here. In some species of crickets, it has been shown that the gut microbiota could provide key enzyme to degrade and assimilate recalcitrant carbohydrates [74, 75]. These findings align with previous studies that reported widespread occurrence of *Wolbachia* in Caelifera [76] but less so in Ensifera [77]. However, the causes of these contrasted colonization patterns remain enigmatic. In the western Swiss Alps, the two host lineages harbor contrasted diet with grasshoppers (Caelifera) being herbivores, while crickets (Ensifera) being more omnivores. We detected diverse lineages of putative gut symbionts including members of the families *Enterobacteriaceae*, *Sphingomonadaceae*, *Streptococcaceae*, and *Erwiniaceae*, notably from the genus *Pantoea*, a widespread bacteria often found in insects [78]. This result is in broad agreement with previous reports of gut microbiota in grasshoppers [75, 79] and crickets [77, 80]. Our finding generalize these results to a unique alpine orthopteran community.

*Wolbachia—*a gram-negative, maternally transmitted bacteria from the order *Rickettsiales—*is probably the most widespread and studied endosymbionts in insects [54] and *Spiroplasma*—an intriguing lineage of wall-less bacteria from the class *Mollicutes*—is exclusively found in hosts (mainly plants and insects) [37, 81]. Accordingly, we found high prevalence and high relative read counts of endosymbiont *Wolbachia* and *Spiroplasma* across samples. The prevalence of both *Wolbachia* and *Spiroplasma* across host species in this study (>75%) is higher than current estimates: 50% of arthropod host species for *Wolbachia* [82] and 7% of western European terrestrial arthropod species for *Spiroplasma* [81]. This suggests that orthopterans may be particularly subject to colonization by both organisms, at least in the Swiss Alps. In agreement with current knowledge, *Wolbachia* ASVs belonged to supergroups A, B, and F that are known to infect arthropods [54], although phylogenetic placement of short 16S sequences comes with high uncertainty. The functional impacts of the endosymbionts on the host remain highly uncertain, especially for population residing in the guts (both in the gut tissue and lumen) [83, 84]. *Wolbachia* is known as a reproductive parasite, it could also be a mutualist in some cases [85]. This is also the case for *Spiroplasma* [81], which can confer resistance against nematodes, parasitoid wasps, and fungi [86]. Our work suggests that both endosymbionts are broadly found in alpine orthopteran, but that more in-depth experimental studies are needed to elucidate their physiological impact on the host.

Future studies could also use full 16S rRNA gene sequencing to better resolve the phylogenetic position of endosymbionts associated with crickets and grasshoppers. Given that endosymbionts like *Wolbachia* are best known to colonize reproductive tissues of many arthropods [54], it is intriguing to recover *Wolbachia* DNA here as we sampled guts, a somatic tissue and not a reproductive tissue. It is increasingly recognized that these symbionts can also colonize cells from somatic tissue, including the gut for *Wolbachia* [83] and the hemolymph for *Spiroplasma* [86]. It is also known that both organisms can colonize in some hosts the lumen compartment of the gut [83, 84, 87]. As we sampled and extracted DNA from the entire gut, it was not possible here to point out the exact tissue location of these endosymbionts. In addition, we cannot fully rule out the possibility that some of the endosymbiont reads originate from the diet ingested by the hosts. Future work using microscopy and/or metabarcoding of more targeted host tissues, for example of gut content versus gut tissue, is needed to elucidate the exact tissue colonized by the endosymbionts. It is unclear whether the dominance of endosymbionts in herbivore grasshoppers has a negative (i.e. parasitic), null (i.e. commensal), or positive (i.e. mutualistic) effect on its host, and further experimental studies are needed to tease apart these different predictions, for example using antibiotic-treated animals. We also note that putative gut symbionts might also be vertically transmitted, as the endosymbionts studied here and it would be interesting for future studies to contrast host specificity to mode of transmission across gut symbionts. For example, one recent study found that more vertical-like transmitted gut microbes from mammals also tend to be more specific to their host, highlighting the importance of transmission for host specificity [17].

In conclusion, our findings were robust to various alternative methods and indicate that, in contrasts to mammals, gut microbiota of orthopteran is less structured by the host identity and host phylogeny and provides a system where phylosymbiosis could be the exception rather than the rule. The higher host specificity observed in endosymbionts corroborates the idea that microbes engaging in vertical transmission are also more specific to their host species.

## Supplementary Material

MS_Supp_Mat_accepted_ycad013

Supp_Table_2_ENA_Submitted_metadata_ycad013

Supp_Table_3_SummarySamleSizes_ycad013

## Data Availability

All DNA data and metadata are available online (microbiome data on ENA: PRJEB62030, host data on figshare: DOI: 10.6084/m9.figshare.23605404). Bioinformatic code is available on GitHub (https://github.com/FloMazel/orthopteran-microbiome).
