# Supplementary material for: Contrasted host specificity of gut and endosymbiont bacterial communities in alpine grasshoppers and crickets
Source: ISME Commun. 2024 Jan 10;4(1):ycad013. doi: 10.1093/ismeco/ycad013 (PMC10875604; doi:10.1093/ismeco/ycad013)
Supplement: MS_Supp_Mat_accepted_ycad013 [file ms_supp_mat_accepted_ycad013.pdf]

Supplementary material for

Contrasted specificity of gut and endosymbiont bacterial communities in alpine grasshoppers and crickets

Affiliations

Mazel, Florent[1,*]

Pitteloud, Camille[2,*]

Guisan, Antoine[1,3,**]

Pellissier, Loïc[4,5,**]

[1]Department of Ecology and Evolution, University of Lausanne, 1015 Lausanne, Switzerland

[2]Département de la mobilité, du territoire et de l'environnement, Service des forêts, de la nature et du paysage, 1950 Sion, Switzerland

[3]Institute of Earth Surface Dynamics, University of Lausanne, 1015 Lausanne, Switzerland

[4]Department of Environmental Systems Science, ETH Zürich, Zürich, Switzerland

[5]Swiss Federal Research Institute WSL, Birmensdorf, Switzerland

*co-first authors

**co-last authors

Supplementary Methods

Reconstruction of the host phylogeny

The host phylogeny relies on sequences of COI, COII, CytB and 16s retrieved from Genbank and completed with COI custom sequencing data and unpublished data from colleagues and (Supp. Table 3). For custom generated sequences, tissue was retrieved from specimens collected for a previous study and stored in EtOH 90% (Pitteloud *et al.* 2022). DNA was extracted from insect leg muscle tissue using a Sbeadex livestock kit following the manufacturer instructions (LGC Genomics, Berlin, Germany). A portion of the COI genetic barcode with a length of 1500 bp was amplified for 7 species using the forward primer UEA1 (gaataattcccataaatagatttaca) and the reverse primer UEA10 (tccaatgcactaatctgccatatta, Lunt *et al.* 1996). The PCR reaction master mix contained the following component concentrations: 1x PCR Gold Buffer without $MgCl_2$ (provided with the Taq), 2 mM $MgCl_2$ (provided with the Taq), 0.2 mM dNTPs, 0.04 U/µl AmpliTaq Gold DNA Polymerase (ThermoFisher, Waltham, MA, USA), and 0.2 µM of each primer (Sigma-Aldrich, St. Louis, MO, USA). Molecular grade water was added to reach 20 µl and 5 µl of DNA extraction product diluted to 2 ng/µl. The PCRs were run under the following conditions: 10 minutes at 95°C; 40 cycles of amplification for 30 seconds at 95°C; 55 seconds at 56°C; 1.5 minutes at 72°C; and 10 minutes at 72°C. PCR products were purified using AMPure (ratio 0.5x, Beckman Coulter, Brea, CA, USA) and sent to Microsynth AG for Sanger sequencing (Balgach, Switzerland). Sequences are available on figshare (DOI: 10.6084/m9.figshare.23605404). Raw sequences were trimmed and paired-end merged in Geneious (Kearse et al., 2012). Sequences were aligned through multiple alignment using a Geneious algorithm (Kearse et al., 2012) with a cost matrix of 93% similarity threshold. Alignments of each marker were concatenated and the phylogeny was generated using the RaxML program (Stamatakis 2014) on the CIPRESS portal (Miller, Pfeiffer, & Schwartz, 2010). *Mantis religiosa* was used as an outgroup. Since *Oedipoda germanica* could not be amplified successfully with PCR, the species was manually added to the tree as a sister species of *Oedipoda caerulescens*. This placement is supported by the high morphological congruence of these two species, which are the only two in the phylogeny belonging to the genus *Oedipoda*. Phylogeny was rooted with the outgoup species *Mantis religiosa.*

We note that in orthopteran taxonomy we used (Baur et al.), some of the named genera are not monophyletic (eg *Chortippus*). In addition, the host phylogeny we present here was constructed with only a few genes so phylogenetic uncertainty is unavoidable and future work should try to reconstruct host phylogenies using as many markers as possible.

Supp. References

Baur B, Roesti C, Thorens P. Grillons et Criquets de Suisse *Hardcover, 352 Pages, Published 2006 by Haupt Verlag. ISBN-13: 978-3-258-07054-4, ISBN: 3-258-07054-7*

Lunt, D.H., Zhang, D.-X., Szymura, J.M. & Hewltt, O.M. (1996). The insect cytochrome oxidase I gene: evolutionary patterns and conserved primers for phylogenetic studies. *Insect Mol. Biol.*, 5, 153–165.

Kearse, M., Moir, R., Wilson, A., Stones-Havas, S., Cheung, M., Sturrock, S., *et al.* (2012). Geneious Basic: An integrated and extendable desktop software platform for the organization and analysis of sequence data. *Bioinformatics*, 28, 1647–9.

Miller, M.A., Pfeiffer, W. & Schwartz, T. (2010). Creating the CIPRES Science Gateway for inference of large phylogenetic trees. In: *2010 Gateway Computing Environments Workshop, GCE 2010*.

Pitteloud, C., Defossez, E., Albouy, C., Descombes, P., Rasmann, S., & Pellissier, L. (2022). DNA-based networks reveal the ecological determinants of plant–herbivore interactions along environmental gradients. *Molecular Ecology, 2022*

Supplementary Tables

	Site	Coordinates.E	Coordinates.N	Elevation	Lat	Long
1	B1	567904	122948	601	46.25719	7.022406
2	B2	568690	124198	743	46.26847	7.032515
3	B3	569913	124765	1056	46.27363	7.048342
4	B4	573582	126076	1384	46.28558	7.095862
5	B5.1	575797	126012	1361	46.28509	7.124606
6	B5.2	574874	126667	1712	46.29094	7.112594
7	B6	574479	124128	1836	46.26809	7.107608
8	B7	578146	125024	2074	46.27628	7.155131
9	B8	578710	124092	2277	46.26791	7.162490

Supplementary Table 1. Sampling site locations. Study site are 100m^2 plots characterized by open semi-natural grasslands distributed along an elevational gradient ranging from 601 m a.s.l to 2277m. Coordinates are given in Swiss coordinate system (LV03) and classical latitude/longitude.

Supplementary Table 2. Sample metadata. See corresponding csv file

Supplementary Table 3. Summary of sample size per sites, host species, and sex. See corresponding csv file

Supplementary Table 4. Host DNA data.
The table provides Genbank accession number and the project ID for custom DNA sequences deposited on ENA for COI, COII, CytB and 16s sequences used to reconstruct the host phylogeny.

	COI	COII	Cytb	16s
Anonconotus alpinus	DOI: 10.6084/m9.figshare.23605404	DOI: 10.6084/m9.figshare.23605404	DOI: 10.6084/m9.figshare.23605404	-
Arcyptera fusca	JN167800	JN187513	AY738384	JF932404
Barbitistes serricauda	AM886788	KF570964	-	AM886635
Bohemanella frigidus	KC261366	KF960030	-	KF729508
Calliptamus italicus	KC261373	DQ099582	DQ366771	FJ555217
Chorthippus apricarius	-	JQ996588	-	-
Chorthippus biguttulus	AY738349	DQ230801	JN187527	-
Chorthippus brunneus	DOI: 10.6084/m9.figshare.23605404	-	-	-
Chorthippus dorsatus	-	DOI: 10.6084/m9.figshare.23605404	DOI: 10.6084/m9.figshare.23605404	-
Chorthippus eisentrauti	DOI: 10.6084/m9.figshare.23605404	-	-	-
Chorthippus mollis	AY738356	-	AY738377	-
Chorthippus parallelus	DOI: 10.6084/m9.figshare.23605404	-	AY738373	JQ580930
Chorthippus vagans	KM384840	-	JN187526	KX426816
Decticus verrucivorus	DOI: 10.6084/m9.figshare.23605404	DOI: 10.6084/m9.figshare.23605404	DOI: 10.6084/m9.figshare.23605404	-
Euthystira brachyptera	AY738367	DQ230796	JN187525	JF932422
Gomphocerippus rufus	NC_014349 *	NC_014349 *	NC_014349 *	Z93285
Gomphocerus sibiricus	GU706074	KT013054	JX122541 *	KX426817
Gryllus campestris	DOI: 10.6084/m9.figshare.23605404	-	AF248662	JX269079
Mecostethus parapleurus	DOI: 10.6084/m9.figshare.23605404	-	-	-
Metrioptera bicolor	EF540830	EF576650	EU120927	EF198433
Metrioptera brachyptera	GU706089	KX429881	-	-
Metrioptera roeselii	JX041527	EF576652	EU120907	EU120763
Metrioptera saussuriana	-	DOI: 10.6084/m9.figshare.23605404	DOI: 10.6084/m9.figshare.23605404	-
Miramella alpina	AF260543	AF227292	AF227291	-
Miramella formosanta	DOI: 10.6084/m9.figshare.23605404	-	-	-
Nemobius sylvestris	-	-	DOI: 10.6084/m9.figshare.23605404	-
Oedipoda caerulescens	EF151835	EF151815	EF151869	KM494859
Oedipoda germanica	-	-	-	-
Omocestus haemorrhoidalis	DOI: 10.6084/m9.figshare.23605404	-	-	KX426832
Omocestus rufipes	DOI: 10.6084/m9.figshare.23605404	DOI: 10.6084/m9.figshare.23605404	-	KF855866
Omocestus viridulus	DOI: 10.6084/m9.figshare.23605404	-	AY738381	KX426837
Phaneroptera falcata	EF540819	EU927344	EF198458	EF198437
Pholidoptera aptera	KY554963	-	-	-
Pholidoptera griseoaptera	DOI: 10.6084/m9.figshare.23605404	-	DOI: 10.6084/m9.figshare.23605404	EU120828
Platycleis albopunctata	EU203991	DOI: 10.6084/m9.figshare.23605404	EU203965	FM882025
Podisma pedestris	AF260542	AY004194	AY738393	-
Polysarcus denticauda	DOI: 10.6084/m9.figshare.23605404	DOI: 10.6084/m9.figshare.23605404	DOI: 10.6084/m9.figshare.23605404	AM886631
Psophus stridulus	EF151843	EF151823	EF151877	-
Ruspolia nitidula	DOI: 10.6084/m9.figshare.23605404	DOI: 10.6084/m9.figshare.23605404	DOI: 10.6084/m9.figshare.23605404	EF198443
Sphingonotus caerulans	GU706137	-	-	KM494793
Stauroderus scalaris	AY738360	JN187518	AY738390	KX426820
Stenobothrus lineatus	FJ555549l	JN002154	JN187529	KX426824
Tetrix bipunctata	KM384855	-	-	-
Tetrix tenuicornis	GU706080	-	AY157556	-
Tettigonia cantans	HM422216	DOI: 10.6084/m9.figshare.23605404	-	EF198429
Tettigonia viridissima	EF540827	EF576646	EU203959	EU120825
Mantis religiosa	FJ802846	FJ806957	-	EF383311

* gene extracted from whole mitochondrial genome

	Factor	Df	Sum Sq	Mean Sq	F value	Pr(>F)	Suborder
1	Species	9	1.665	0.185	2.903	0.006	Ensifera
2	Sex	1	0.152	0.152	2.383	0.128	Ensifera
3	Species:Sex	8	0.363	0.045	0.712	0.680	Ensifera
4	Residuals	62	3.950	0.064	NA	NA	Ensifera
5	Species	13	4.572	0.352	6.350	0.000	Caelifera
6	Sex	1	0.113	0.113	2.040	0.155	Caelifera
7	Species:Sex	12	1.344	0.112	2.023	0.023	Caelifera
8	Residuals	228	12.629	0.055	NA	NA	Caelifera

Supplementary Table 5. Effect of host species and Sex on endosymbiont relative read count. Table depicts the results of ANOVA tests for different subset of the data.

	Predictor	Df	SumOfSqs	R2	F	Pr(>F)	Symbiont
1	Elevation	1	0.200	0.009	1.158	0.301	Endosymbiont
2	Sex	1	0.102	0.004	0.598	0.770	Endosymbiont
3	Species	14	14.681	0.639	6.178	0.001	Endosymbiont
4	Elevation	1	0.442	0.018	1.086	0.302	Putattive gut symbiont
5	Sex	1	0.419	0.017	1.045	0.350	Putattive gut symbiont
6	Species	13	7.359	0.298	1.402	0.001	Putattive gut symbiont

Supplementary Table 6. PERMANOVA results on balanced dataset for endosymbionts and putative gut symbionts. Table depict mean statistics (n=100 repetition) of PERMANOVA models (Bray Curtis metric) run for endosymbionts and putative gut symbionts for balanced sampling (4 individuals in 15 species).

Supplementary Figures

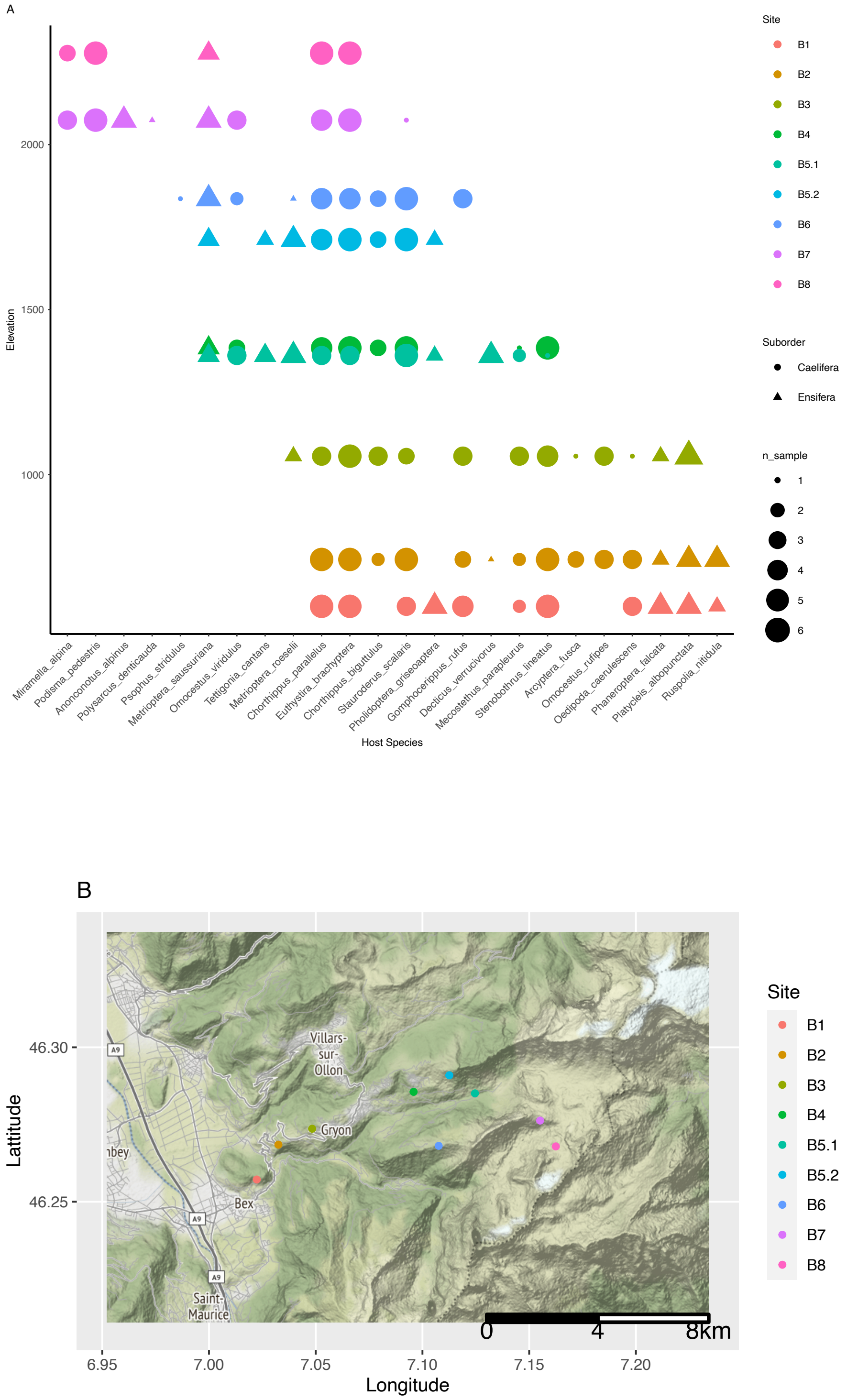

Supplementary Figure 1. Sampling campaign design. A) The plot illustrates the distribution of samples from different host species in different sampling sites along the elevational gradient. B) Map of the sampling sites.

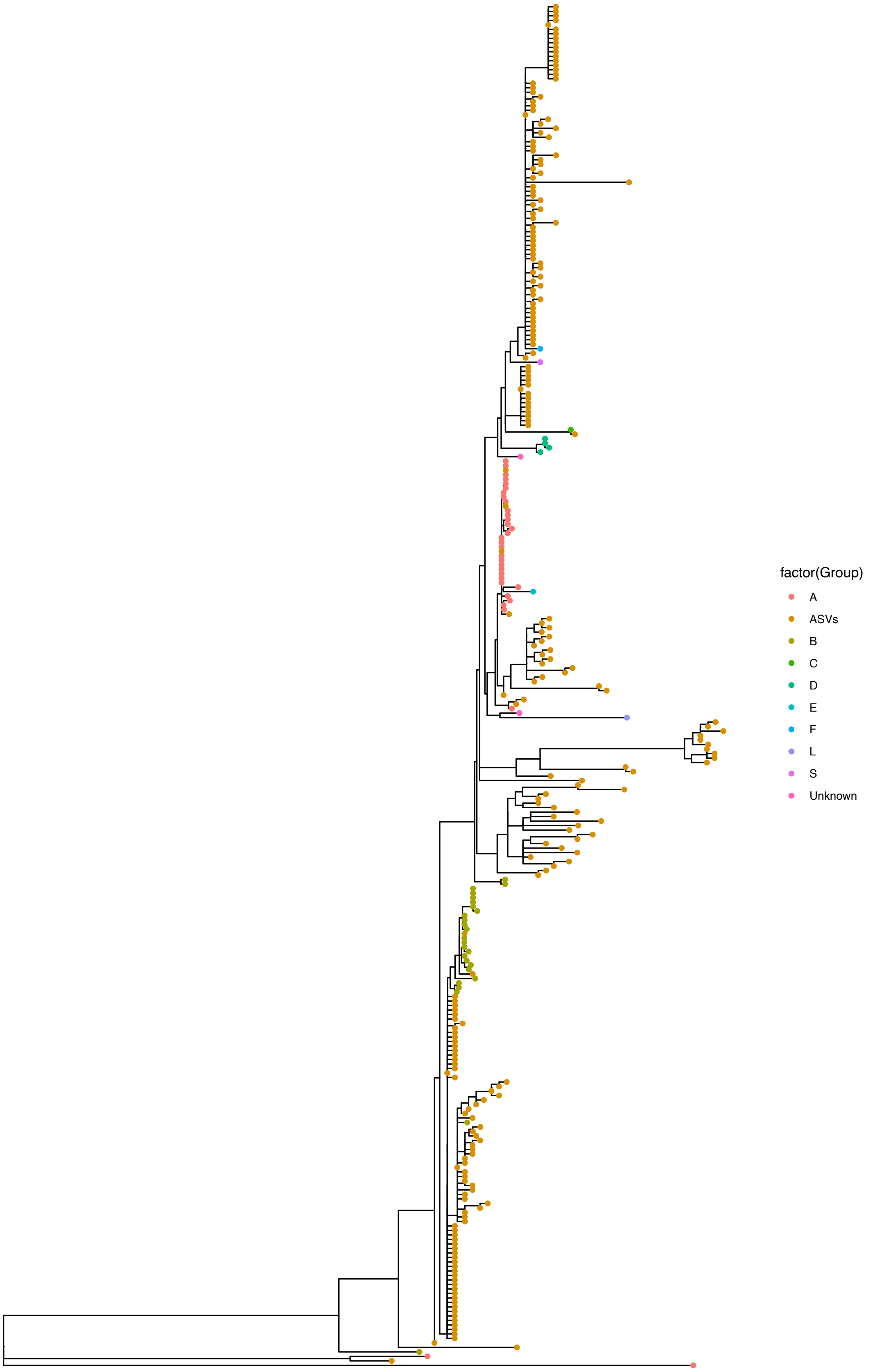

Supplementary Figure 2. Wolbachia phylogeny. Phylogenies of ASVs inferred from partial 16S sequences using a backbone phylogeny using representative Wolbachia full 16S sequences. ASVs from the metabarcoding data is represented in brown while full 16S sequences from genome assemblies are represented with the other colors (see legend).

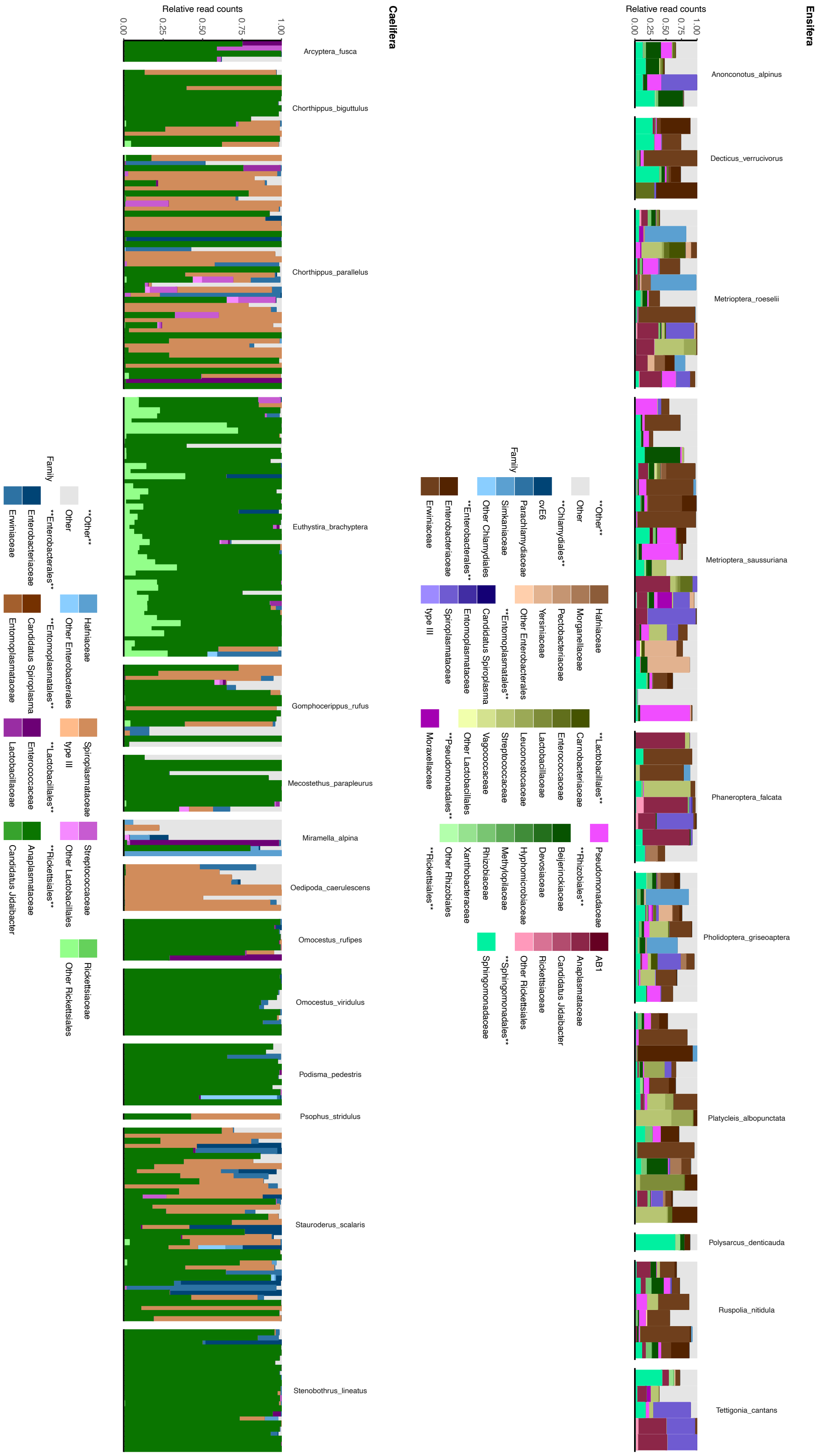

Supplementary Figure 3. Taxonomic composition of the samples. The plot depicts the relative read counts (Y-axis) of the samples (X-axis) for both Caelifera (right panel) and Ensifera (left panel). Taxonomic composition at the Family level (within Orders) is provided.

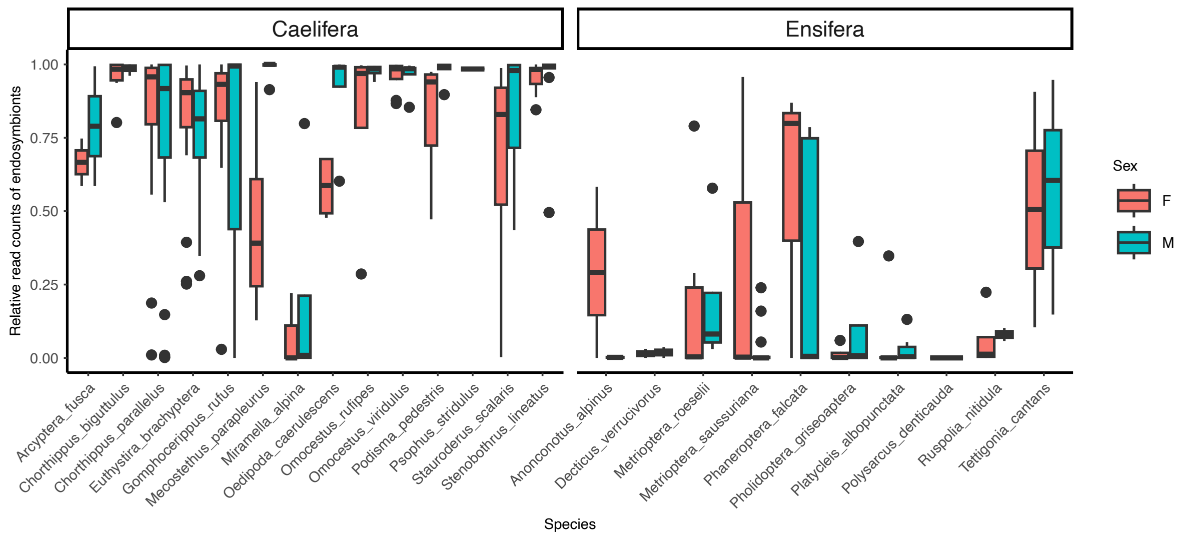

Supplementary Figure 4. Relative read counts of endosymbionts in different species and different sex. The plot depicts the relative read counts (Y-axis) of the samples in different host species and sex (X-axis). The lower and upper hinges correspond to the first and third quartiles (the 25th and 75th percentiles). The upper whisker extends from the hinge to the largest value no further than 1.5 * IQR from the hinge (where IQR is the inter-quartile range, or distance between the first and third quartiles). The lower whisker extends from the hinge to the smallest value at most 1.5 * IQR of the hinge. Data beyond the end of the whiskers are called "outlier" points and are plotted individually.

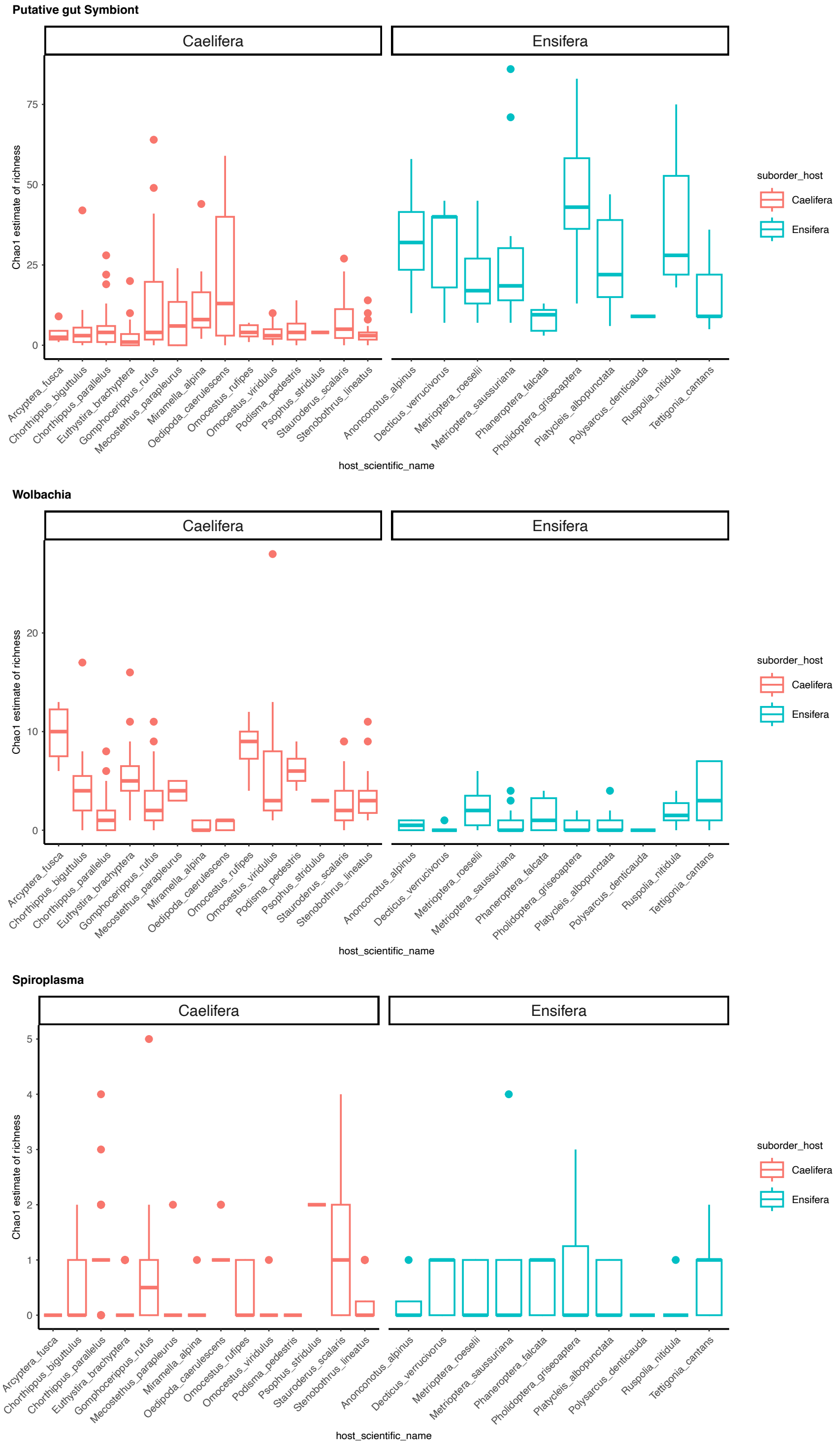

Supplementary Figure 5. Alpha-diversity estimates. The plot depicts alpha diversity (Y-axis, Chao1 metric) of the samples in different host species (X-axis). The lower and upper hinges correspond to the first and third quartiles (the 25th and 75th percentiles). The upper whisker extends from the hinge to the largest value no further than 1.5 * IQR from the hinge (where IQR is the inter-quartile range, or distance between the first and third quartiles). The lower whisker extends from the hinge to the smallest value at most 1.5 * IQR of the hinge. Data beyond the end of the whiskers are called "outlier" points and are plotted individually.

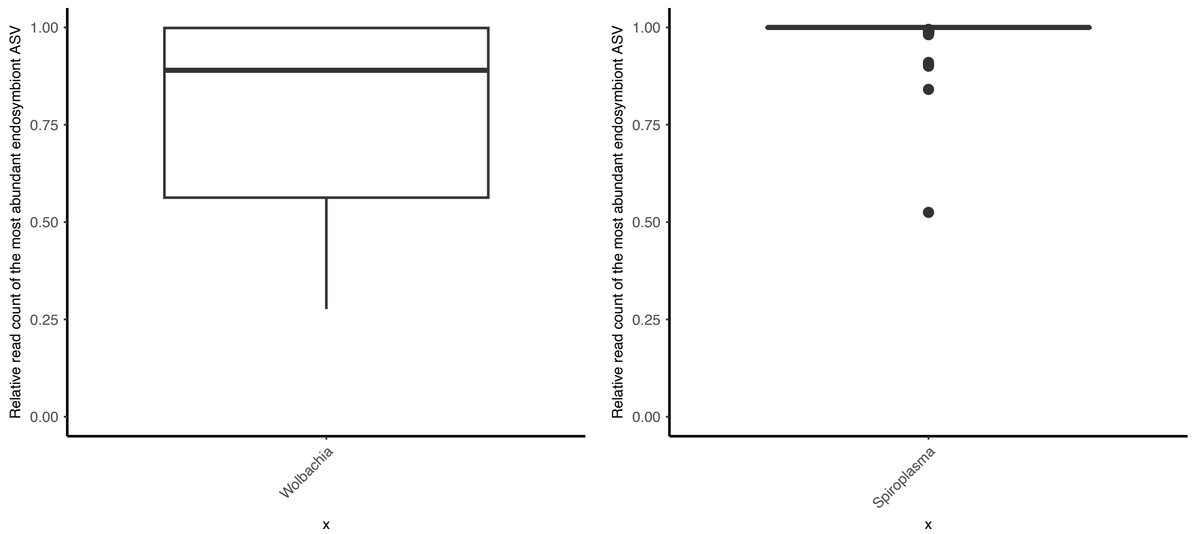

Supplementary Figure 6. Relative read counts of the most dominant endosymbiont ASV. The plot depicts the distribution of the relative read counts (Y-axis) of the dominant ASV in each sample samples for different endosymbionts (left and right panels). The lower and upper hinges correspond to the first and third quartiles (the 25th and 75th percentiles). The upper whisker extends from the hinge to the largest value no further than 1.5 * IQR from the hinge (where IQR is the inter-quartile range, or distance between the first and third quartiles). The lower whisker extends from the hinge to the smallest value at most 1.5 * IQR of the hinge. Data beyond the end of the whiskers are called "outlying" points and are plotted individually.

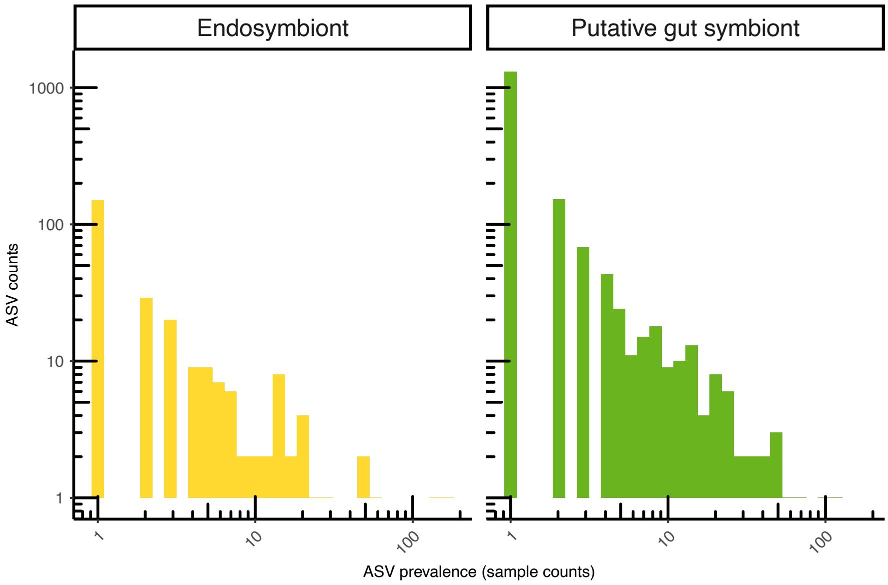

Supplementary Figure 7. ASV prevalence across samples.

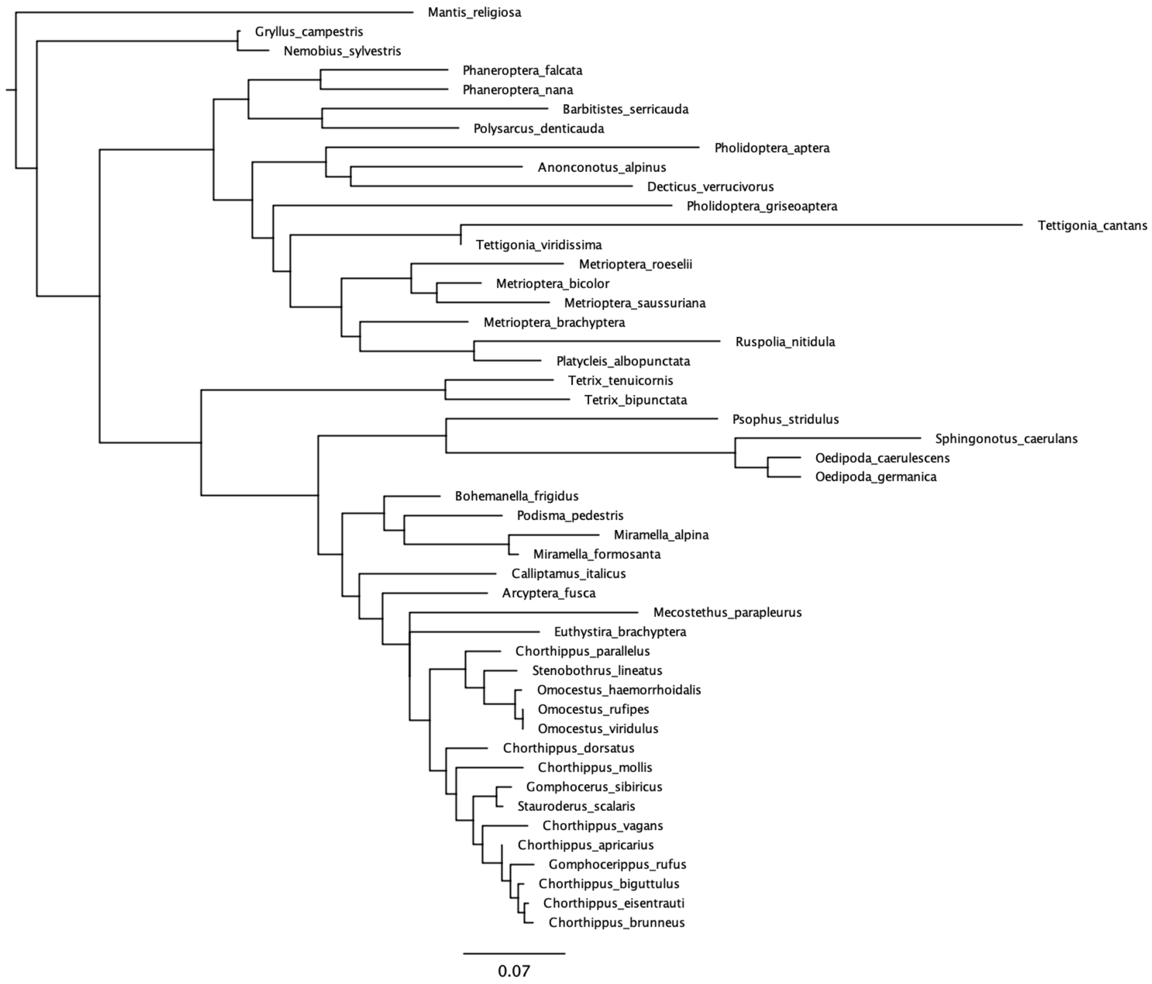

Supplementary Figure 8. The host phylogeny. See methods for details.

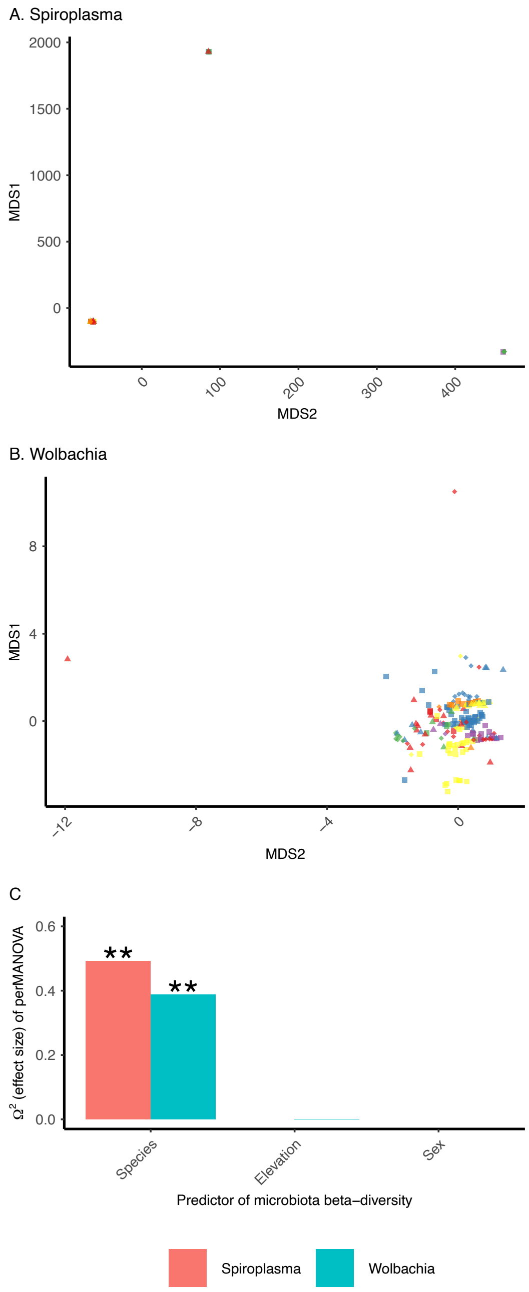

Supplementary Figure 9. Species specificity effect within *Wolbachia* and *Spiroplasma* communities. The figure illustrates (panels A-B) and report statistical measures (panel C) of host specificity. Panel A-B are multidimensional representations of microbiome composition (NMDS axes based on Bray-Curtis dissimilarities between samples) for ***Spiroplasma*** (panel A) and ***Wolbachia*** (panel B). Panel C depicts the strength of the effect (Y-axis) of different host factors (X-axis) on microbiota composition (PERMANOVA model on beta-diversity). The “host species” effect measures the strength of host specificity at the species level. The asterisk refers to the level of significance of the corresponding factor in the PERMANOVA models

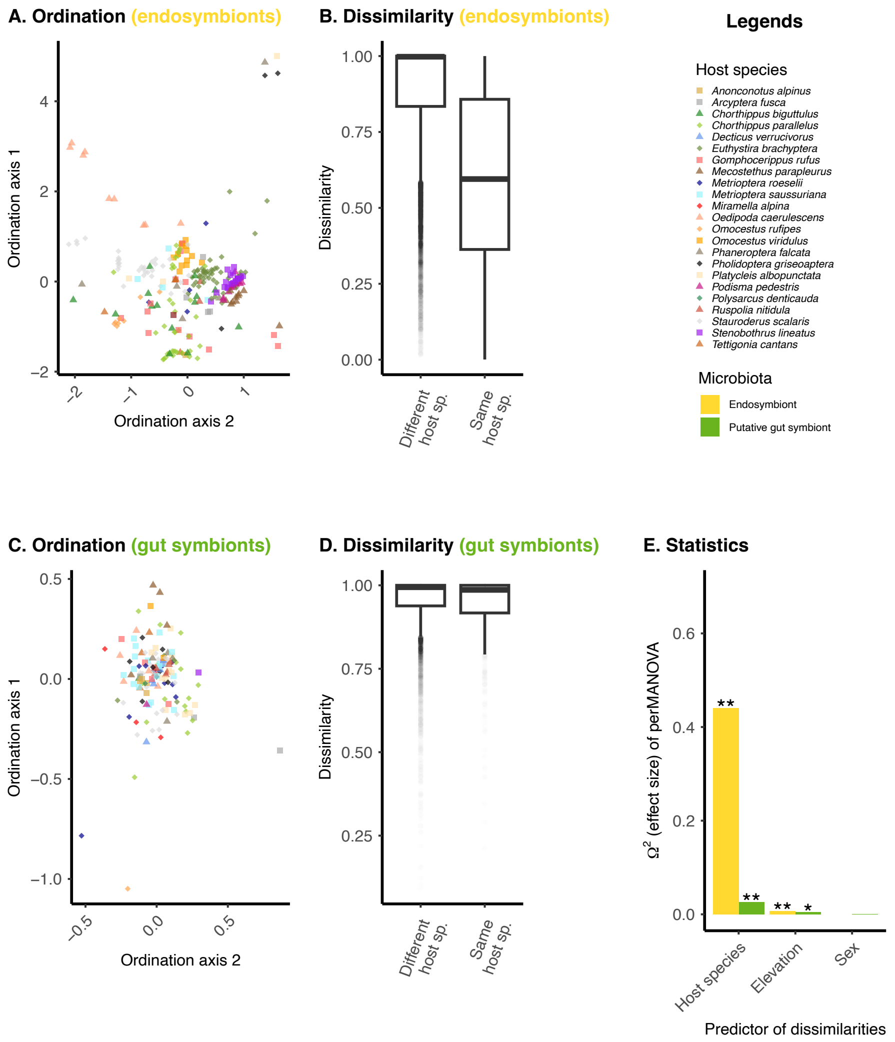

Supplementary Figure 10. Beta-diversity analysis on unrarefied data. The figure illustrates (panels A-B) and report statistical measures (panel C) of host specificity. Panel A-B are multidimensional representations of microbiome composition (NMDS axes based on Bray-Curtis dissimilarities between samples) for endosymbiont (panel A) and gut symbiont communities (panel B). Panel C depicts the strength of the effect (Y-axis) of different host factors (X-axis) on microbiota composition (PERMANOVA model on beta-diversity). The "host species" effect measures the strength of host specificity at the species level. The asterisk refers to the level of significance of the corresponding factor in the PERMANOVA models

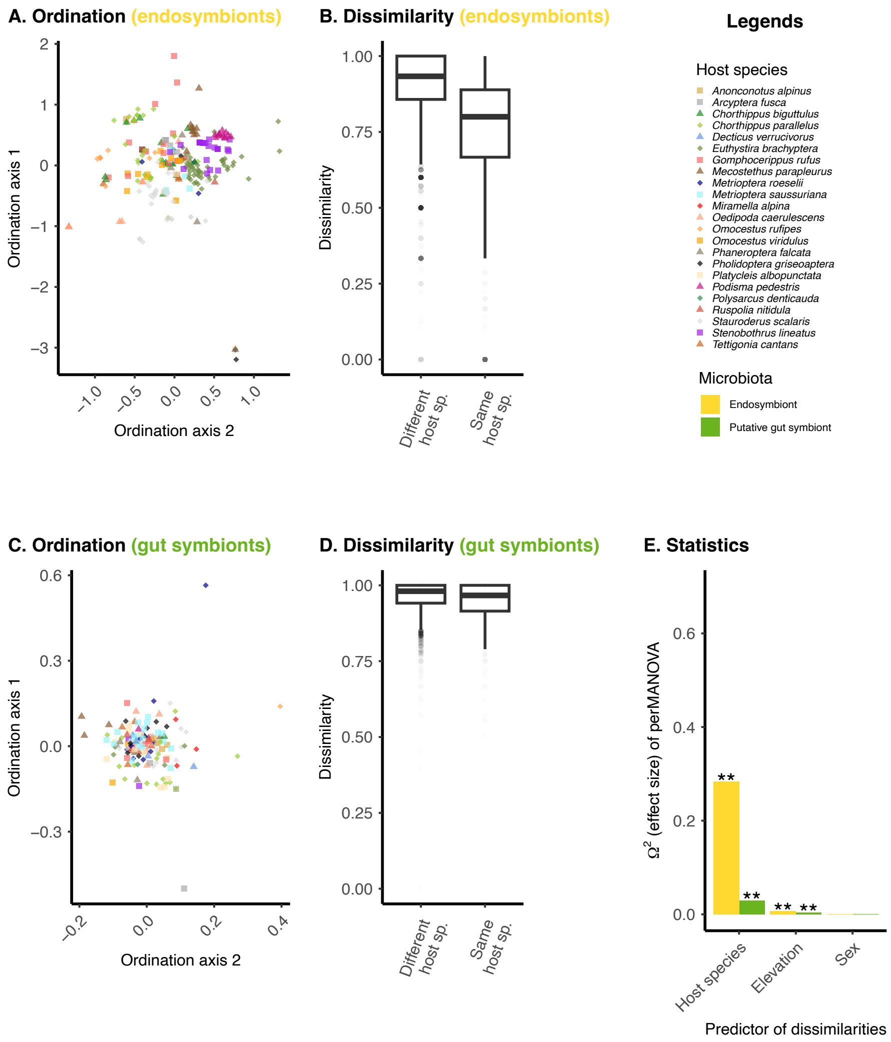

Supplementary Figure 11. Beta-diversity analysis with Jaccard beta-diversity metric. The figure illustrates (panels A-B) and report statistical measures (panel C) of host specificity. Panel A-B are multidimensional representations of microbiome composition (NMDS axes based on Jaccard dissimilarities between samples) for endosymbiont (panel A) and gut symbiont communities (panel B). Panel C depicts the strength of the effect (Y-axis) of different host factors (X-axis) on microbiota composition (PERMANOVA model on beta-diversity). The “host species” effect measures the strength of host specificity at the species level. The asterisk refers to the level of significance of the corresponding factor in the PERMANOVA models.

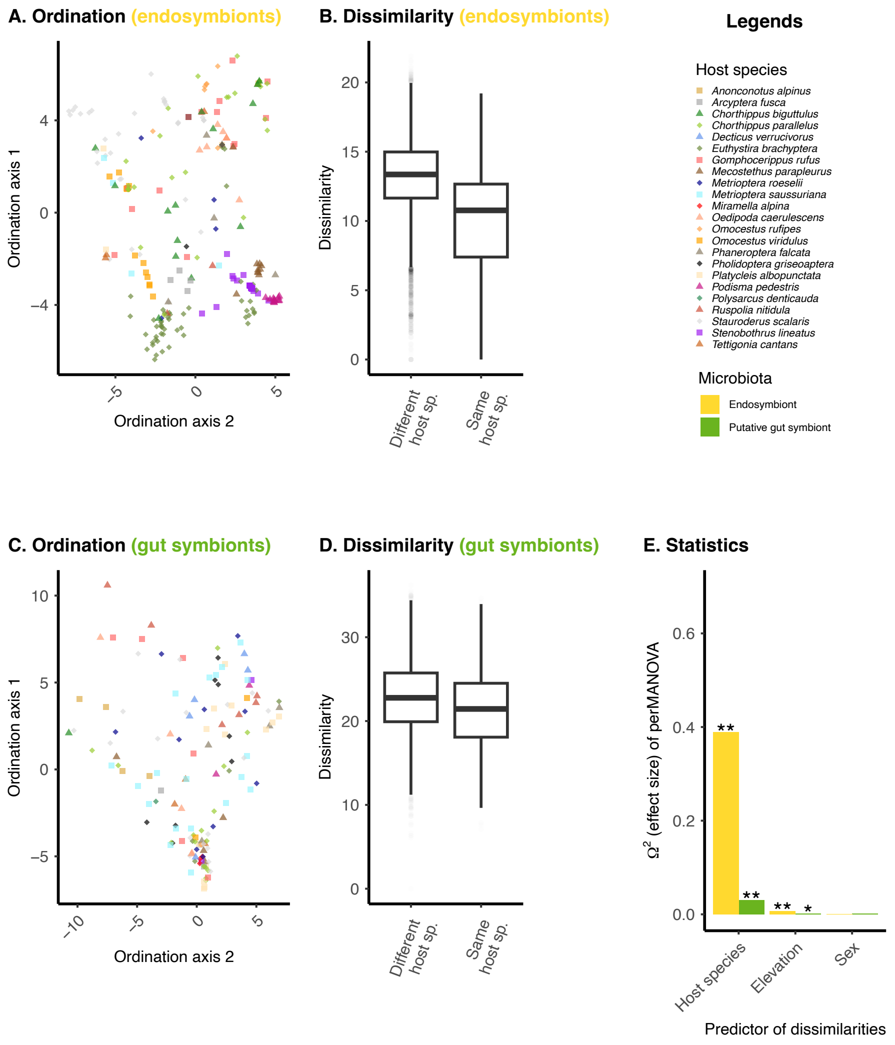

Supplementary Figure 12. Beta-diversity analysis with Aitchison beta-diversity metric. The figure illustrates (panels A-B) and report statistical measures (panel C) of host specificity. Panel A-B are multidimensional representations of microbiome composition (PCoA axes based on Aitchison dissimilarities between samples) for endosymbiont (panel A) and gut symbiont communities (panel B). Panel C depicts the strength of the effect (Y-axis) of different host factors (X-axis) on microbiota composition (PERMANOVA model on beta-diversity). The "host species" effect measures the strength of host specificity at the species level. The asterisk refers to the level of significance of the corresponding factor in the PERMANOVA models

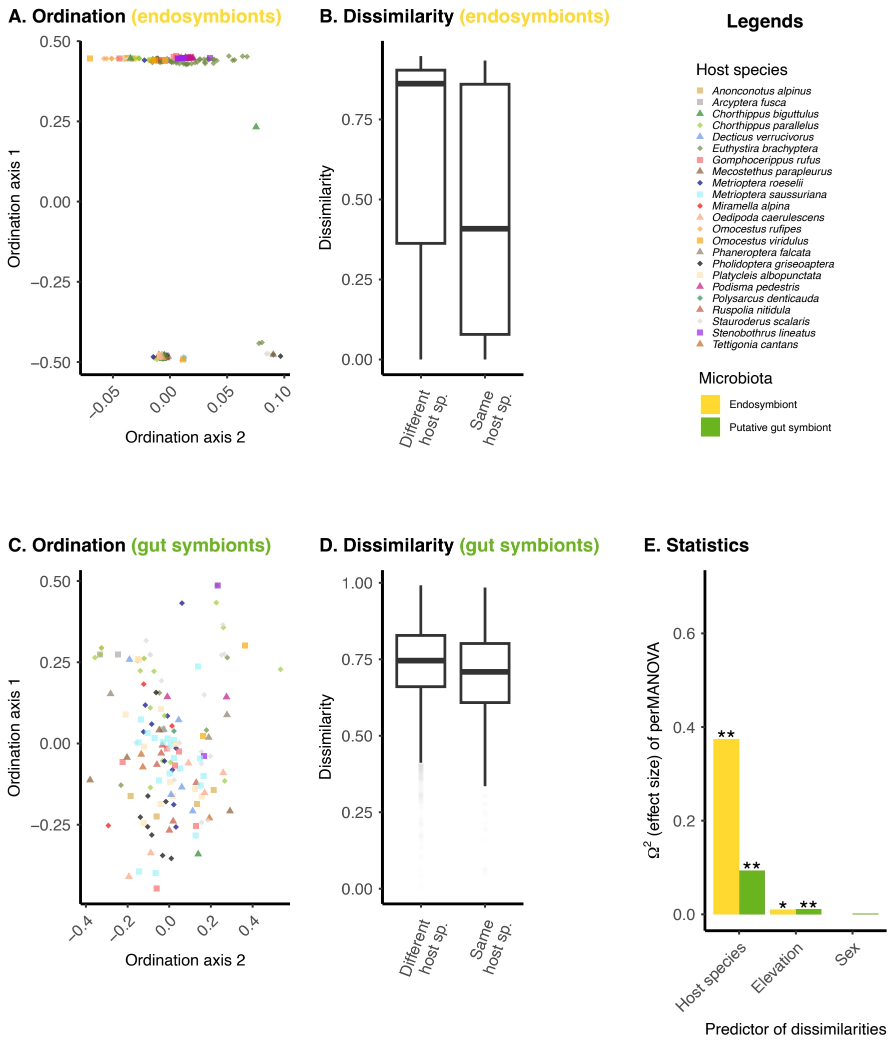

Supplementary Figure 13. Beta-diversity analysis with Unifrac beta-diversity metric. The figure illustrates (panels A-B) and report statistical measures (panel C) of host specificity. Panel A-B are multidimensional representations of microbiome composition (NMDS axes based on Unifrac dissimilarities between samples) for endosymbiont (panel A) and gut symbiont communities (panel B). Panel C depicts the strength of the effect (Y-axis) of different host factors (X-axis) on microbiota composition (PERMANOVA model on beta-diversity). The "host species" effect measures the strength of host specificity at the species level. The asterisk refers to the level of significance of the corresponding factor in the PERMANOVA models.

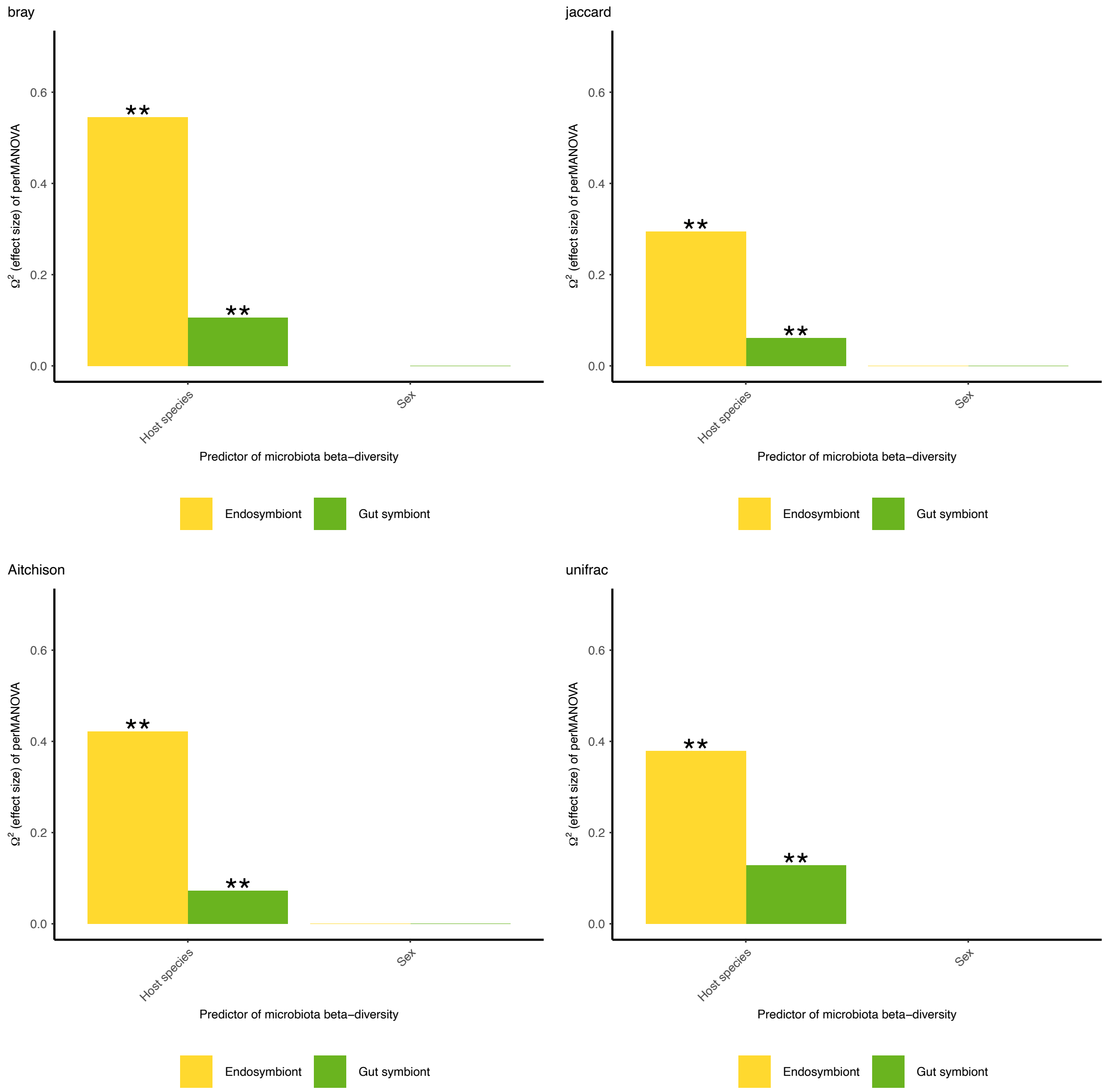

Supplementary Figure 14. Constrained permutation procedure confirms Permanova results. The figure depicts the strength of the effect (Y-axis) of different host factors (X-axis) on microbiota composition (PERMANOVA model on beta-diversity) when permutation are constrained by sampling sites. The “host species” effect measures the strength of host specificity at the species level. The asterisk refers to the level of significance of the corresponding factor in the PERMANOVA models. Different panel corresponds to different beta-diversity metrics.

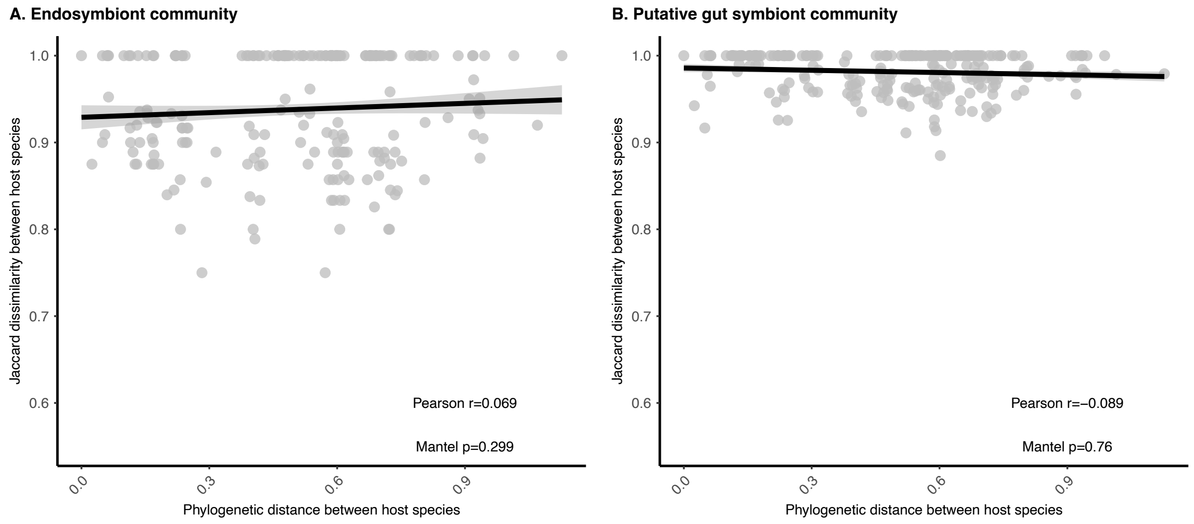

Supplementary Figure 15. Host phylogenetic specificity of endosymbiont and gut symbiont communities. The figure depicts the relationship between microbiota dissimilarity (Jaccard measure) and host phylogenetic distance (X-axis) for endosymbionts (panel A) and gut symbiont (panel B) communities. Mantel p-value based on 999 permutations.

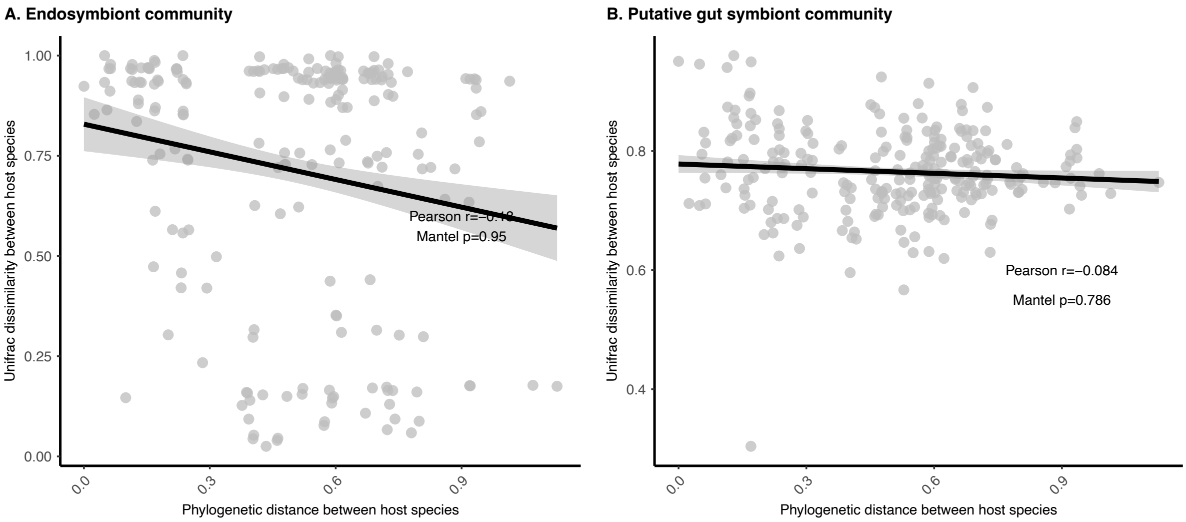

Supplementary Figure 16. Host phylogenetic specificity of endosymbiont and gut symbiont communities (Unifrac). The figure depicts the relationship between microbiota dissimilarity (Unifrac measure) and host phylogenetic distance (X-axis) for endosymbionts (panel A) and gut symbiont (panel B) communities. Mantel p-value based on 999 permutations.

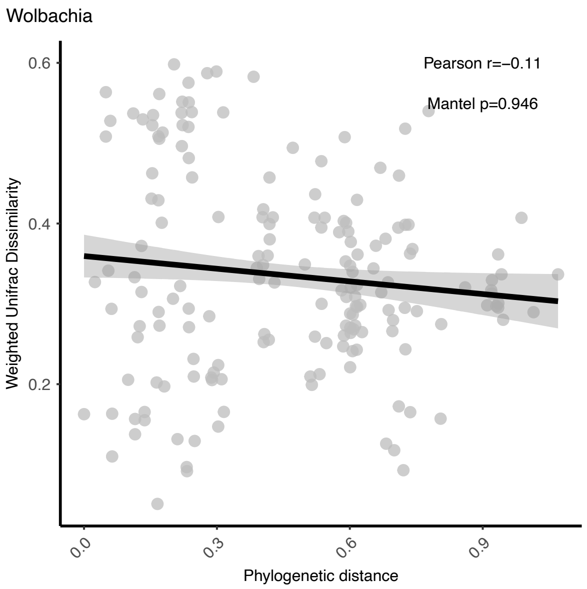

Supplementary Figure 17. Host phylogenetic specificity of endosymbiont and gut symbiont communities. The figure depicts the relationship between microbiota dissimilarity (weighted Unifrac measure) and host phylogenetic distance (X-axis) for Wolbachia. Mantel p-value based on 999 permutations. Unifrac is based on the phylogeny inferred for Wolbachia sequence only (see methods).

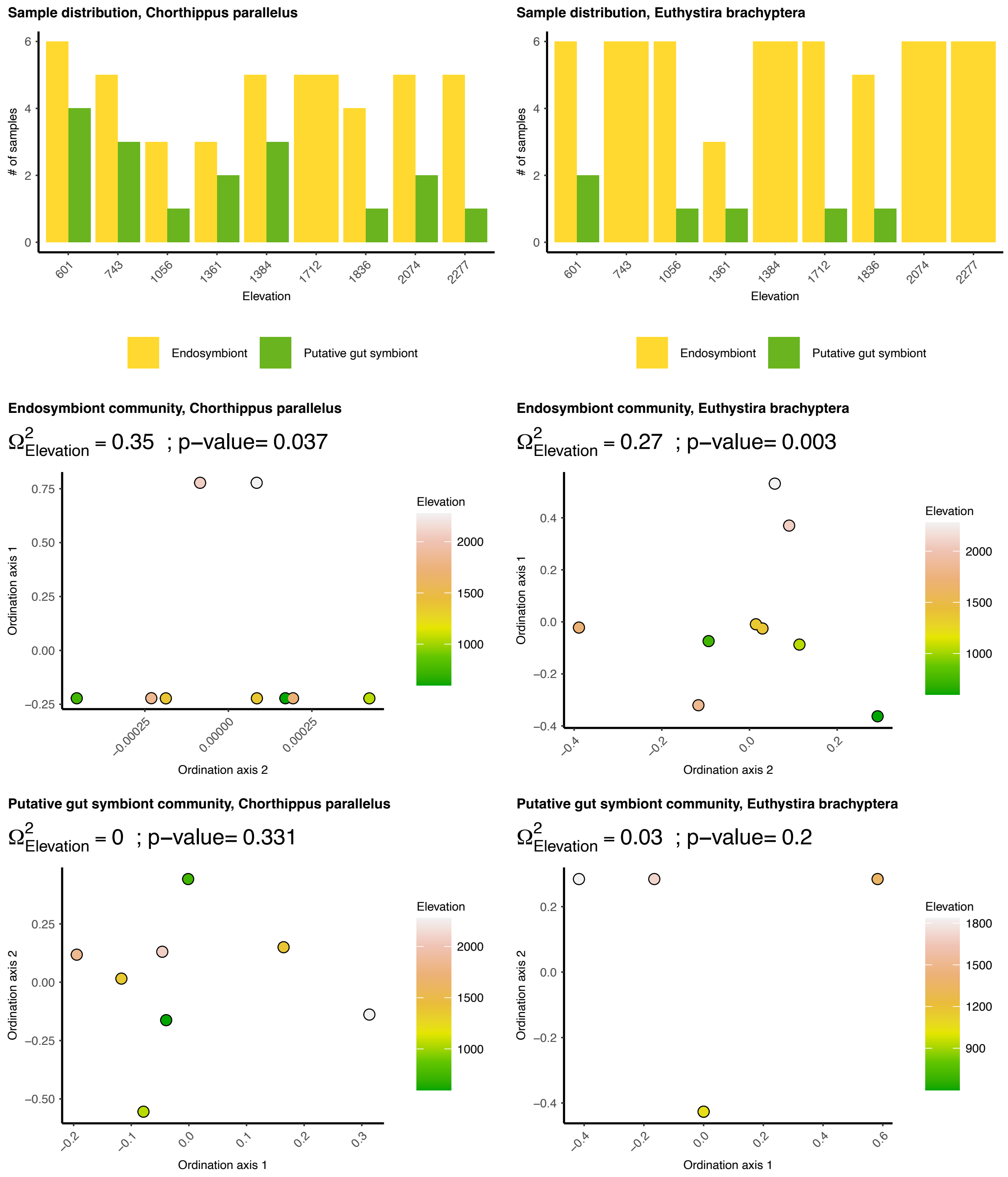

Supplementary Figure 18. Effect of elevation on Beta-diversity analysis within two host species with Bray-Curtis metric. For two host species (columns), the figure illustrates sample distribution across the elevational gradient (top panels) and ordinations of composition (endosymbionts: middle panels; putative gut symbionts: lower panels) along with PERMANOVA test of elevational effects (subtitles). Dissimilarities between sites have been averaged (see methods).

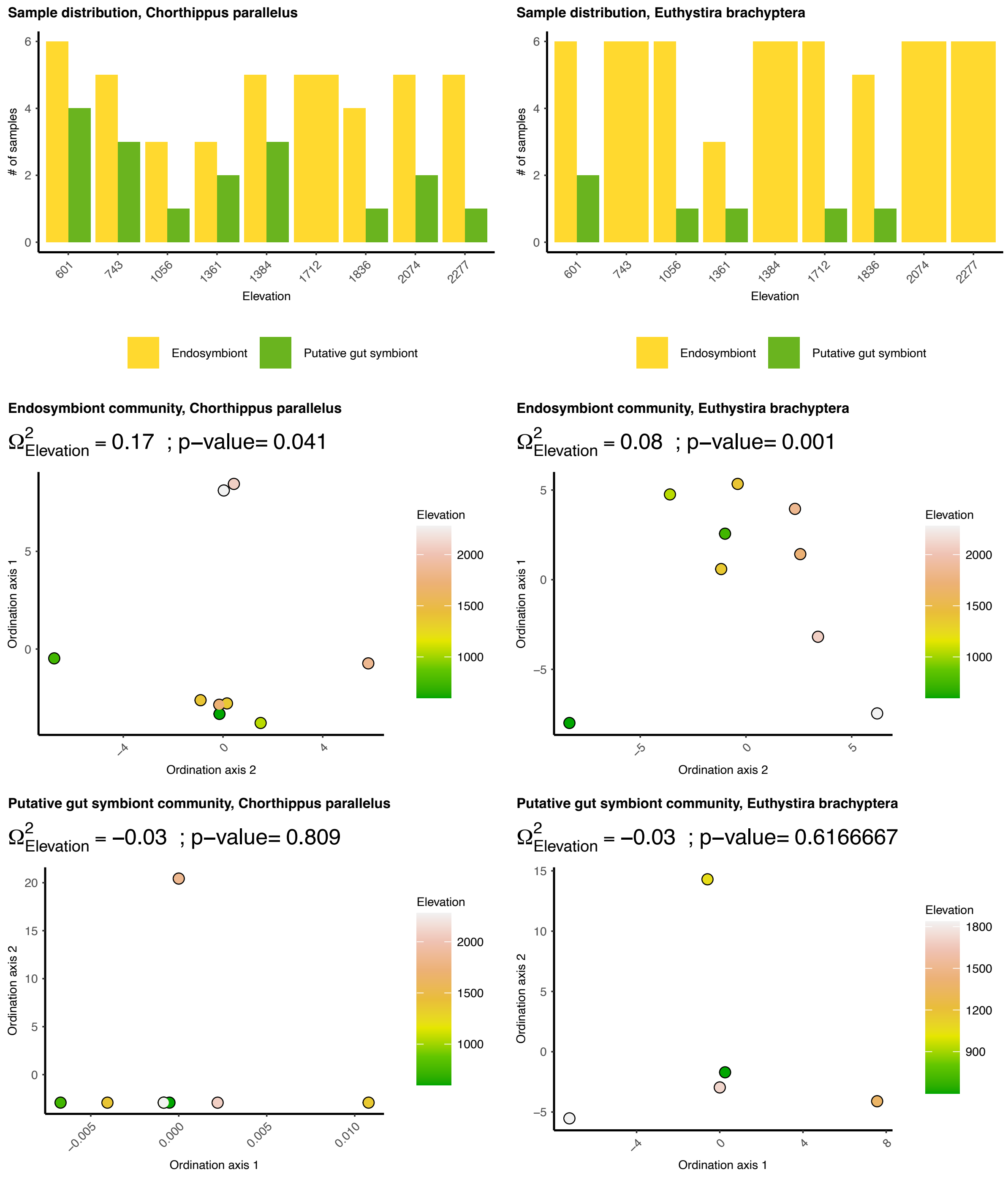

Supplementary Figure 19. Effect of elevation on Beta-diversity analysis within two host species with Aitchison metric. For two host species (columns), the figure illustrates sample distribution across the elevational gradient (top panels) and ordinations of composition (endosymbionts: middle panels; putative gut symbionts: lower panels) along with PERMANOVA test of elevational effects (subtitles). Dissimilarities between sites have been averaged (see methods).

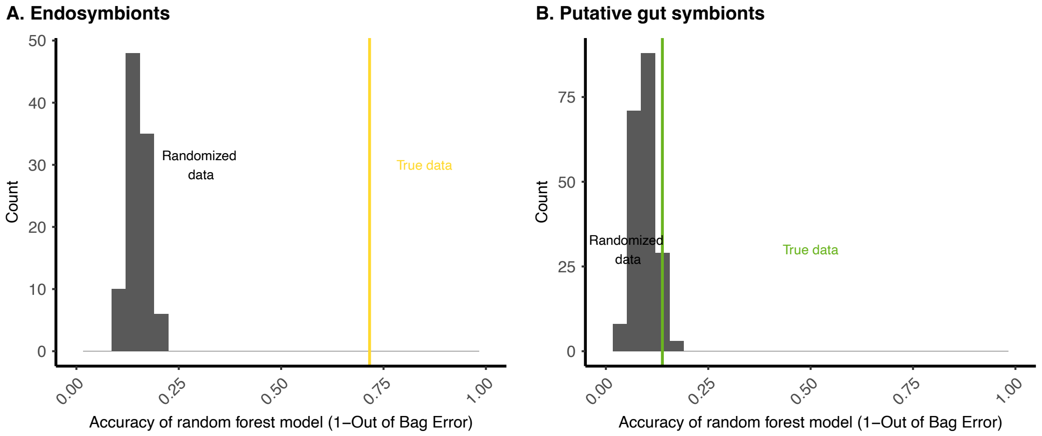

Supplementary Figure 20. Results of random forest models to classify host species identity from ASV distribution across samples. Observed error of the model are shown by the vertical line in color and the error of the models with randomized (n=100) host identity are show as histogram.
